# Immunogenic T cell epitopes of SARS-CoV-2 are recognized by circulating memory and naïve CD8 T cells of unexposed individuals

**DOI:** 10.1016/j.ebiom.2021.103610

**Published:** 2021-10-06

**Authors:** Isaac Quiros-Fernandez, Mansour Poorebrahim, Elham Fakhr, Angel Cid-Arregui

**Affiliations:** Targeted Tumor Vaccines Group, Clinical Cooperation Unit Applied Tumor Immunity, German Cancer Research Center (DKFZ), Im Neuenheimer Feld 280, 69120 Heidelberg, Germany

**Keywords:** SARS-CoV-2, COVID-19, T cell epitopes, pre-existing cross-reactivity, CD8 memory T cells, immunotherapy, vaccine

## Abstract

**Background:**

Recent studies have provided evidence of T cell reactivity to Severe Acute Respiratory Syndrome Coronavirus 2 (SARS-CoV-2) in significant numbers of non-infected individuals, which has been attributed to cross-reactive CD4 memory T cells from previous exposure to seasonal coronaviruses. Less evidence of cross-reactive memory CD8 T cells has been documented to date.

**Methods:**

We used the NetCTLPan neural network of the Epitope Database and Analysis Resource to select a series of 27 HLA-A*02:01 epitopes derived from the proteome of SARS-CoV-2. Their binding capacity was assessed by a HLA-A*02:01 stabilization assay and by quantifying their binding to HLA-A*02:01 monomers for the generation of tetramers. Their ability to stimulate and induce expansion of SARS-CoV-2 reactive CD8 T cells was measured by flow cytometry. The TCR repertoire of COVID convalescent and healthy unexposed donors was analysed using the MIRA database.

**Findings:**

The HLA-A*02:01 epitopes tested were able to stabilise HLA molecules and induce activation of CD8 T cells of healthy unexposed donors. Our results, based on specific tetramer binding, provide evidence supporting the presence of frequent cross-reactive CD8 T cells to SARS-CoV-2 antigens in non-exposed individuals. Interestingly, the reactive cells were distributed into naïve, memory and effector subsets.

**Interpretation:**

Our data are consistent with a significant proportion of the reactive CD8 T clones belonging to the public shared repertoire, readily available in absence of previous contact with closely related coronaviruses. Furthermore, we demonstrate the immunogenic capacity of long peptides carrying T cell epitopes, which can serve to isolate virus-specific T cell receptors among the ample repertoire of healthy unexposed subjects and could have application in COVID-19 immunotherapy. Limitations of our study are that it concentrated on one MHC I allele (HLA-A*02:01) and the low numbers of samples and epitopes tested.

**Research in context**

**Evidence before this study**

SARS-CoV-2 is an emerging virus that causes the current COVID-19 pandemic. Emergency vaccines based on the spike protein of the virus have been developed and are currently being administered. Cytotoxic CD8 T cells are critical for clearing viral infection. However, the nature of the T cell responses toward this virus and its implications in the pathogenesis of COVID-19 are still unclear. Previous studies on healthy unexposed individuals have reported mainly CD4 T cells reactive to SARS-CoV-2. Nevertheless, the pre-existence of reactive CD8 T cells in the unexposed unvaccinated population and its implications are still controversial and need detailed investigation.

**Added value of this study**

Using a series of peptides derived from the viral proteome this study demonstrates that unexposed-unvaccinated individuals carry a significant fraction of circulating CD8 T cells reactive to epitopes of various SARS-CoV-2 proteins. These reactive cells are distributed between memory and naïve T cells. Interestingly, the frequencies of SARS-CoV-2-specific CD8 T cell receptor clonotypes reactive to virus antigens in 88 healthy unexposed donors were comparable to those of 31 matched convalescent patients. Overall, our results suggest that the public shared repertoire contains an unexpected fraction of CD8 T cell receptor clonotypes that can readily react to different viral epitopes including those of the spike (S), replicase and N proteins.

**Implications of all the available evidence**

The efficiency of cellular immune responses after infection with SARS-CoV-2 are ultimately linked to the availability and frequency of T cell clones that are reactive to viral epitopes. Our study provides evidence indicating that the CD8 T cell repertoire contains significant, albeit variable frequencies of virus-reactive clones in the peripheral blood of unexposed-unvaccinated individuals. Given the fact that a substantial proportion of them are naïve T cells and that T cell epitopes are well conserved among SARS-CoV-2 variants, this study supports the notion that pan-coronavirus immunity can be achieved by incorporating T cell target epitopes like those characterized here in next generation vaccines. Further studies are needed with larger numbers of unexposed donors, vaccinated and convalescent subjects and patients with different HLA alleles.

Alt-text: Unlabelled box

## 1 Introduction

Since December 2019 the severe acute respiratory syndrome coronavirus 2 (SARS-CoV-2) has spread throughout the world causing severe pneumonia with rapid progression known as coronavirus disease-2019 (COVID-19), a pandemic with substantial mortality for which there is no specific therapy. So far, clinical studies with nucleotide analogues, such as Remdesivir, antibody combinations or protease inhibitors have shown variable outcomes. Several strategies applying available prophylactic vaccines targeting the S glycoprotein are expected to control the pandemic in the near future [1,2]. Nevertheless, the continued emergence of variants of concern makes necessary the search for new treatments and vaccines, which requires a better understanding of the B and T cell immune responses prior and during infection.

B cell epitopes derived from SARS-CoV proteins, principally the S protein, have shown therapeutic efficacy [3]. Human neutralizing monoclonal antibodies (NAbs) targeting the receptor-binding domain (RBD) of the S protein of SARS-CoV-2 have been isolated from COVID-19 patients at acute phase [4], and it is believed that Nab responses are essential for infection control and survival of critically ill patients [5]. Further, protection from symptomatic SARS-CoV-2 infection correlates with Nab levels [6]. Clinical trials employing convalescent plasma have shown reduction in the mortality rate among COVID-19 patients who were not receiving mechanical ventilation [7]. However, the impact of NAbs along the COVID-19 course remains still controversial with some reports suggesting an unfavourable correlation of Nabs with disease progression [8,9]. Recently, a study using convalescent plasma was halted because no effect was observed in emergency department patients with mild symptoms [10].

T cell-specific epitopes are gaining particular attention in diagnosis, prevention, and treatment of COVID-19 [11]. It has been suggested that T cells might be responsible for immunity against SARS-CoV-2 in individuals who have not been exposed to SARS-CoV-2 [12]. Although the distinct mechanisms of pre-existing reactivity are not fully understood, it has been attributed to cross-reactivity against circulating "common cold" coronaviruses, particularly OC43, HKU1, NL-63, and 229E and, in regions where it is endemic, SARS-CoV [[13], [14], [15]]. Nevertheless, the role of B cells and cross-neutralizing antibodies targeting SARS-CoV-2 epitopes in protecting unexposed individuals appears to be minimal [16,17]. Individuals unexposed to SARS-CoV-2 very rarely have neutralizing antibodies against the RBD of the S protein [[18], [19], [20]]. Moreover, the presence of antibodies against seasonal coronaviruses does not seem to prevent SARS-CoV-2 infection in children [21].

Several studies have consistently reported SARS-CoV-2 cross-reactive memory CD4+ T cells in 28%-50% of unexposed individuals [12,13,22,23]. However, the presence and significance of cross-reactive memory CD8+ T cells remains speculative [24]. Here, we selected and characterized the immunogenic potential of a series of HLA-A*02:01 CD8 T cell epitopes from the SARS-CoV-2 proteome. This study demonstrates several immunogenic epitopes of SARS-CoV-2 and provides evidence supporting the existence of memory and naïve cross-reactive CD8 T cells in unexposed individuals. Furthermore, using long synthetic peptides (LSPs) we show that the epitopes are properly processed and presented by dendritic cells (DCs) and are able to activate autologous CD8 T cells. These results suggest that LSPs carrying SARS-CoV-2 epitopes could be of use as vaccines and in immunotherapy of COVID-19.

## 2 Material and Methods

### 2.1 Cell culture condition and cytokines

PBMCs were cultured in X-VIVO™ 20 medium (Lonza, Walkersville, MD, USA). T2 cell line was cultured in RPMI 1640 (Gibco) with 10% fetal bovine serum (FBS; Gibco). IL1β, IL-2, IL-4, IL-6, IL-15, TNFα and GM-CSF in carrier-free state were obtained from Biolegend (San Diego, CA, USA).

### 2.2 Antibodies

Anti-HLA-A*02 APC (RRID AB 2561567, clone BB7.2), anti-CD3 PerCPCy5.5 (RRID AB 2561628, clone OKT3) and PE (RRID AB 571913, clone OKT3), anti-CD8 FITC (RRID AB 1877178, clone SK1), anti-CD137 PECy7 (RRID AB 2207741, clone 4B4-1), anti-CD45RO PECy7 (RRID AB 11203903, clone UCHL1), anti-CD45RA PerCPCy5.5 (RRID AB 893358, clone HI100), anti-CCR7 APC-Cy7 (RRID AB 10915272, clone G043H7) were bought from Biolegend (San Diego, CA, USA).

### 2.3 Peptides

Short peptides (8-11 amino acid) were obtained from ProteoGenix (Strasbourg, France) with a purity about 90%. The peptides were dissolved in pure DMSO to a concentration of 20 mM and stored at -80°C. The long synthetic peptides (LSPs) were designed by adding eight amino acids to each side of the short peptides, identical in sequence to the original SARS-CoV-2 proteins. Additionally, we incorporated a 9-mer arginine tail in the C-terminal to facilitate penetration of the long peptide into dendritic cells. They were also obtained from ProteoGenix with purity about 90%. The LSPs were dissolved in DMSO to a concentration of 5 mM and were stored at -80°C.

### 2.4 *In silico* analysis

For the in silico prediction of possible HLA-A*02:01 restricted SARS-CoV-2 epitopes, we introduced the protein sequences of the virus in the NetCTLPan neural network model from the Immune Epitope Database and Analysis Resource [25]. This model offers a possibility to separately predict proteasome degradation, TAP processing, and HLA binding scores given a specific protein and HLA molecule [26]. Then, we proceeded to select 27 peptides with high combined scores for proteasome and TAP processing, as well as high HLA-A*02:01 affinity. We further estimated the binding affinity of each peptide to HLA-A*02:01 by docking studies. Molecular docking between the epitope library and HLA-A*02:01 was conducted by DockTope [27] and HLA-Arena [28]. The binding energy (ΔG) of HLA-A*02:01/epitope complexes was calculated using the PRODIGY server [29].

In order to identify shared epitopes between SARS-CoV-2 and other human coronaviruses, we introduced the respective protein sequences in the NetCTLPan neural network model looking for HLA-A*02:01-restricted epitopes, then we selected the epitopes with overall higher similarity to those of SARS-CoV-2. Additionally, we generated a similarity matrix using pairwise sequence alignment. The whole proteome of SARS-CoV, MERS, OC43, HKU1, NL-63, and 229E were obtained from GeneBank. We defined a model for similarity calculation in which the score 3 was considered for identical residues (shown by '*' sign in sequence alignments), 2 for highly similar residues (shown by ':' sign in sequence alignments), 1 for relatively similar (shown by '.' sign in sequence alignments), and 0 for completely different residues. The final accumulative values were converted to the percentage and represented in heatmap graphs.

### 2.5 Isolation of PBMCs

Peripheral blood of healthy donors was collected prior to March 2019, before the onset of SARS-CoV-2. Blood was first diluted 1:2 with PBS+2 mM EDTA and then centrifuged over Ficoll® Paque Plus (GE Healthcare) following the recommended protocol by the provider. The PBMCs were frozen in aliquots of 4-6 × 10^7^ cells in freezing medium (90% FBS and 10% DMSO) and stored in liquid nitrogen. We tested all donor samples used in the study for antibodies against SARS-CoV-2 (Covid IgG IgM Test Kit, WuHan UNScience Biotechnology) and by RT-PCR and, as expected, all samples were negative.

### 2.6 Dendritic cell differentiation and activation

We cultured 1 × 10^7^ PBMCs for 2 hours at 37°C in one well of a six-well plate, the non-adherent cells were removed with two washes using culture medium. The adherent cells were left in culture for five days in X-VIVO™ 20 medium containing IL-4 (25 ng/mL) and GM-CSF (25 ng/mL), refreshed when it started to turn yellow. Afterwards this medium was replaced with activation medium: X-VIVO™ 20 supplemented with TNFα (20 ng/mL), IL1β (20 ng/mL) and IL6 (20 ng/mL). After 48h, the cells were retrieved, centrifuged and used for co-culture with the peptides and subsequently the T cells. Flow cytometry gating and analysis of dendritic cells is exemplified in the supplementary data (Fig S1).

### 2.7 HLA-A*02:01 stabilization assay (shift assay)

In a 96-well plate 5 × 10^4^ T2 cells were seeded in 50 µL of RPMI 1640 without serum. Immediately, 50 µL of a 2x peptide solution was added to the well to reach a final peptide concentration of 100 µM. The cells were then incubated for 18h at 37°C. Then, the cells were harvested, washed with FACS buffer (PBS pH=7.2 containing 0.5% bovine serum albumin) and stained with anti-HLA-A2 antibody (1 µg/mL). T2 cells incubated with no peptide, with just vehicle (DMSO 0.5%), or with the EBV EBNA3B 416-424 (IVTDFSVIK) peptide served as a negative control. The pp65 495-503 (NLVPMVATV) strong HLA-A2 binder peptide of CMV was used as a positive control. All peptides and conditions were tested in triplicate. Three independent tests were performed for each peptide.

### 2.8 Generation of tetramers

To generate peptide-loaded tetramers we used Flex-T™ HLA-A*02:01 UVX monomers (Biolegend) for peptide exchange by UV radiation according to the instructions of the manufacturer. In brief, 20 µL of the HLA-A*02:01 UVX monomer were mixed with 20 µL of the respective SARS-CoV-2 peptide diluted to 400 µM in PBS. Then, the mixture was UV irradiated (366 nm) for 30 min, as recommended by the manufacturer. The irradiated solutions were immediately incubated at 37°C for additional 30 min. The efficiency of peptide exchange was measured by ELISA using 2 µL of the reaction, the rest of the reaction volume was mixed with APC-streptavidin (Biolegend) to generate the tetramers. Finally, we added D-biotin to the tetramer solution to block any remaining free APC-streptavidin. The tetramers were always prepared the day before of the staining, and left at 4°C in the dark overnight.

### 2.9 Ultraviolet peptide exchange evaluation

Briefly, ELISA plates were coated with a streptavidin solution (2 µg/mL, Biolegend) overnight, afterwards, we blocked unspecific binding for 30 min using the binding buffer, next we added 100 µL of the Flex-T HLA-A*02:01 reactions diluted 1:1400 in the dilution buffer, and incubated the plate for 1 hour at 37°C. Then we added 100 µL of the diluted anti-β2 microglobulin HRP-conjugated antibody (0.3 µg/mL, Biolegend) to each well and incubated the plate for 1 hour at 37°C. Next, we added 100 µL of the substrate solution and after 8 min at room temperature on a plate shaker at 400, we added 50 µL of the stop solution (2% oxalic acid dehydrate, Sigma) and measured the absorbance at 405 nm in a Perkin Elmer Victor™ X4 2030 Multilabel Reader. Each peptide exchange efficiency was evaluated in three independent experiments, we included a negative control using the EBV EBNA3B 416-424 (IVTDFSVIK) peptide and a positive control using the CMV pp65 495-503 (NLVPMVATV) peptide. We calculated the peptide exchange efficiency as percentile of the signal obtained with test peptide against the positive control peptide.

### 2.10 Measurement of IFN-γ secretion and CD137 expression

These assays were performed in triplicate using X-VIVO™20 medium (Lonza) supplemented with IL-2 (10 ng/mL), IL-15 (10 ng/mL) and POM-1 (20 µM). POM-1 (polyoxometalate-1) enhances CD8 T cell activation by inhibiting CD39/ENTPD-1 (ecto-nucleoside triphosphate diphosphohydrolase-1), the rate-limiting enzyme in the hydrolysis of extracellular ATP [30]. First, 5 × 10^4^ DCs were seeded in 50 µL of medium per well of a 96-well plate. Then, we added 50 µL of medium containing 30 µM of the respective peptide and incubated the cells at 37°C/5%CO2 for one hour. The following controls were included: (i) no peptide; (ii) vehicle (0.05%DMSO); and (iii) an HLA-A*02:01 CMV peptide (pp65 495-503: NLVPMVATV). Subsequently, 5 × 10^5^ cells of autologous PBMCs were added to each well in 50 µL of medium (final volume per well = 150 µL). The co-cultures were maintained for 7 days, replacing half of the medium with fresh medium when the colour turned yellow (every 1-2 days). At day 7, a re-stimulation was performed by adding 10 µM of the respective peptide and incubation was continued for 3 hours. The cells were then retrieved and analysed for activation using the IFN-γ-Catch Assay (Miltenyi Biotech) following the manufacturer instructions. In brief, the PBMCs were washed once with FACS Buffer and resuspended in 50 µL of ice cold IFN-γ Catch Reagent diluted 1:10 in X-VIVO™20 medium. After 5 minutes of incubation on ice, 500 µL of pre-warmed (37°C) medium were added and the samples were incubated for 45 min at 37°C in a tube rotator. The cells were washed with FACS buffer and stained with anti-CD3-PerCPCy5, anti-CD8-FITC and the IFN-γ Detection Antibody-PE (diluted 1:10 in FACS Buffer) for 30 min on ice. Finally, the cells were washed twice with FACS buffer and analysed by flow cytometry. All samples were analysed using a BD FACSCanto II™ flow cytometer.

Alternatively, after peptide re-stimulation, the DCs/PBMCs were co-incubated further 18 hours at 37°C/5%CO2 to allow CD137 upregulation take place in the activated cells, since CD137 has a more delayed response compared to IFN-γ secretion. Then, the cells were retrieved, washed once with FACS Buffer and stained with anti-CD3-PerCPCy5, anti-CD8-FITC and anti-CD137-PECy7 for 30 min on ice, washed twice and analyzed in a BD FACSCanto II™. Gating of IFN-γ+ or CD137+/CD3+CD8 T cells was performed as exemplified in the supplementary information (Fig S2). Dead cells were excluded by staining with DAPI.

### 2.11 Detection of T cells reactive to SARS-CoV-2 peptides

PBMCs from HLA-A*02:01 positive donors unexposed to SARS-CoV-2 were used to isolate CD3+CD8 T cells using CD8+ human T cell isolation kit (Miltenyi Biotech). The mean recovery rate of CD8+ T cells after isolation, measured by flow cytometry, was 91.6% (± 5%). The isolated cells were cultured overnight in X-VIVO™ 20 medium supplemented with IL-2 (10 ng/mL) and IL-15 (10 ng/mL). Then, the protein kinase inhibitor Dasatinib (Cayman Chemicals), which has been shown to enhance the binding of fluorochrome-conjugated peptide-major histocompatibility complex (pHLA) tetramers [31] was added to the culture medium to a final concentration of 50 nM, and the cells were further incubated for 30 min at 37°C. Afterwards, the cells were retrieved and washed with cold FACS buffer, and subsequently resuspended in FACS buffer containing the respective pHLA-tetramers (2 µL of the pHLA-tetramer stock in 100 µL of FACS buffer per 10^6^ CD3+CD8+ T cells) and incubated on ice in the dark for 30 min. Then, the cells were washed twice with FACS buffer and stained with anti-CD3-PE, anti-CD8-FITC, anti-CD45RO-PECy7, anti-CD45RA-PerCPCy5.5 and anti-CCR7-APCCy7 antibodies for 30 min on ice in the dark. To compensate for unspecific binding, we used tetramers bearing the HLA-A*02:01-restricted epitope HLVEALYLV from insulin (Ins 10-18) peptide as control (Fig S3). Staining was performed under the same conditions as with tetramers carrying SARS-CoV-2 peptides. The frequencies of unspecific tetramer+ cells observed for the different naïve and memory CD8 T cell subpopulations were subtracted accordingly. Additionally, to further validate the specificity of the binding of the pHLA A*02:01 tetramers, we conducted a staining of a non-HLA-A*02:01 donor and three HLA A*02:01 donors, using the ins 10-18 tetramers alongside each of the SARS-CoV-2 peptide tetramers (Fig S4). Finally, the cells were washed and resuspended in FACS buffer containing DAPI (0.5 µg/mL). All samples were acquired and analysed using a BD FACSCanto II™.

### 2.12 Expansion of SARS-COV-2 reactive CD8 T cells by peptide stimulation

Mature dendritic cells (mDCs) were loaded with either a short peptide (P, 10 µM) or the corresponding long peptide (LSP, 2.5 µM) for three hours at 37°C. Then, we added autologous PBMCs (5 × 10^5^ PBMCs to 1 × 10^5^ DCs), following this ratio (5:1) for all samples. The proportion of CD3**^+^**CD8**^+^** T cells in the PBMCs varied from donor to donor representing between 25% and 45% of the total number of cells. Throughout the co-culture the peptides were maintained in the medium, which consisted of X-VIVO™ 20 supplemented with IL-2 (10 ng/mL) and IL-15 (10 ng/mL). We replaced half of the medium every other day and added fresh cytokines and peptide. At day 7 of co-culture, the cells were first treated for 30 min with Dasatinib (50 nM) and then retrieved, washed and stained for pHLA-tetramer-APC. Afterwards, the cells were washed with FACS buffer and stained with anti-CD3-PE, anti-CD8-FITC, anti-CD45RO-PECy7, anti-CD45RA-PerCPCy5.5 and anti-CCR7-APCCy7 antibodies for 30 min on ice in the dark. Finally, the cells were washed and resuspended in FACS buffer containing DAPI (0.5 µg/mL). All samples were acquired and analysed using a BD FACSCanto II™.

### 2.13 Analysis of TCR β repertoire from unexposed and COVID convalescent donors

For bioinformatic analyses of the TCR-β repertoire, we used the ImmuneCODE™ database, which contains the datasets for over 1400 SARS COV-2 exposed individuals [32], and a healthy unexposed control database comprised of 88 individuals from a pre-COVID-19 sampling [33].

For the analysis of the TCR repertoire of patients and unexposed individuals we used the ImmunoSEQ Analyzer 3.0 of Adaptive Biotechnologies. First, we focused in the MIRA database, which comprises a TCR-β dataset of COVID-19 convalescent patients whose PBMCs were re-stimulated with pools of SARS-CoV-2 peptides, sorted based on enhanced expression of the CD137 activation marker and subsequently sequenced [32].

We filtered in the HLA-A*02:01 MIRA database donors and further focused only on the samples stimulated with peptide pools containing one of our selected peptides (P3, P12, P16 and P21). However, P16 was not included in any peptide pool and hence it was excluded from this analysis.

Then, we classified and counted the clones that were identical at the complementarity determining region 3 (CDR3) of the TCR-β genes reactive to the selected pools. Next, we selected the three most abundant clones reactive to each peptide pool and analyzed their frequencies in the repertoire databases of recovered patients (using as tags “Dataset-COVID-19-Adaptive” and “Category-Recovered”) as well as in the unexposed database (using the tag “Epidemiological status: Healthy”).

### 2.14 Ethics statement

All samples used in this study were from healthy donors and were obtained from the Blood Banking Facilities (Institut für Klinische Transfusionsmedizin und Zelltherapie Heidelberg, IKTZ, gGmbH), where all healthy control participants provided informed consent and authorization and permission for their blood being used for scientific research. We received de-identified blood bags after being processed by apheresis. It is not possible for us to trace any data from the donors (age, gender or any other). The blood samples used in this study were received between March 2018 and February 2019, i.e. before SARS-CoV-2 appearance and spreading. The datasets of clonotypes of HLA-A2 SARS-CoV-2 convalescent patients and healthy donors were from Adaptive Biotechnologies and were also de-identified.

### 2.15 Statistical analysis

We used the GraphPad Prism software version 9.0.0 to perform statistical analyses. Normally distributed samples were compared using a one-way ANOVA test, considering p≤ 0.05 as significant. Moreover, not normally distributed samples, like medians of TCR clonotype frequencies, were compared by applying a Mann-Whitney-Wilcoxon test, again considering p≤ 0.05 as significant. Error bars were used to represent the standard deviation (SD) obtained from the inter-experimental replicates.

### 2.16 Role of the funding source

The funder of the study had no role in the study design, data collection, analysis, interpretation nor writing of the manuscript.

## 3 Results

### 3.1 Identification of HLA-A*02:01-restricted CD8 T cell epitopes derived from SARS-CoV-2 proteins

We first sought to select a representative panel of HLA-A*02:01-restricted peptides derived from SARS-CoV-2 proteins, which could be CD8 T cell epitopes. To this end, we used the NetCTLpan T cell epitope prediction tool as implemented in the Proteasomal cleavage/TAP transport/MHC class I combined predictor resource in the Immune Epitope Database and Analysis Resource (IEDB) [[34], [35], [36], [37]]. For the purpose of this study, we focused on the search of relevant CD8 T cell epitopes considering the probability of being processed, transported and presented on HLA-A*02:01, one of the most prevalent and studied MHC class I molecules. The amino acid sequences of relevant SARS-CoV-2 proteins (Uniprot entries P0DTC2, P0DTD1, P0DTC9, P0DTC5 and P0DTC4) were analysed with the IEDB tools to categorize peptides of 8-11 amino acids according to their combined scores. Among them we selected for further characterization a series of 27 peptides (P1 to P27, Table I) with the highest combined scores regarding proteasome and TAP processing and HLA-A2 binding. We chose peptides within the viral proteome as follows: S-protein (P1-P6); replicase pp1ab (P7-P16); N (P17-P18); 3a (P19-P22); E (P23-P24); and M (P25-P27).

**Table 1:** Predicted HLA-A*02:01 restricted epitopes derived from six SARS-CoV-2 proteins. The # indicates the epitope starting amino acid within the protein sequence. The predicted scores are directly proportional to the binding and processing of the peptides.

SARS-CoV-2 protein	No.	#	Sequence	MHC binding score	TAP score	Cleavage score	Combined score
S	1	269	YLQPRTFLL	0.875	0.892	0.977	1.117
	2	976	VLNDILSRL	0.741	0.996	0.970	0.984
	3	691	SIIAYTMSL	0.774	1.156	0.951	1.017
	4	417	KIADYNYKL	0.727	1.177	0.966	0.973
	5	1060	VVFLHVTYV	0.751	0.597	0.812	0.948
	6	1192	NLNESLIDL	0.612	1.018	0.945	0.850
pp1ab	7	3183	FLLNKEMYL	0.887	0.907	0.945	1.122
	8	1675	YLATALLTL	0.851	0.964	0.946	1.088
	9	84	VMVELVAEL	0.805	1.193	0.957	1.050
	10	3732	SMWALIISV	0.878	0.670	0.956	1.110
	11	3085	FLMSFTVL	0.898	0.992	0.951	1.136
	12	4515	TMADLVYAL	0.884	1.020	0.965	1.126
	13	5246	LMIERFVSL	0.859	1.159	0.974	1.107
	14	6246	LLADKFPVL	0.830	1.149	0.968	1.076
	15	6425	MMISAGFSL	0.849	1.333	0.801	1.062
	16	7003	FLIGCNYL	0.890	1.102	0.963	1.134
N	17	222	LLLDRLNQL	0.816	1.044	0.976	1.061
	18	112	YLGTGPEAGL	0.656	0.739	0.679	0.827
3a	19	107	YLYALVYFL	0.929	1.189	0.580	1.089
	20	45	WLIVGVALL	0.702	0.986	0.945	0.939
	21	139	LLYDANYFL	0.906	1.020	0.961	1.147
	22	72	ALSKGVHFV	0.868	0.522	0.930	1.090
E	23	20	FLAFVVFLL	0.906	0.944	0.891	1.130
	24	50	SLVKPSFYV	0.828	0.512	0.951	1.055
M	25	28	FLTWICLL	0.804	0.891	0.902	1.029
	26	26	FLFLTWICLL	0.798	0.961	0.902	1.025
	27	92	WLSYFIASFRL	0.759	0.979	0.972	1.002

### 3.2 SARS-CoV-2 epitopes efficiently stabilize HLA-A*02:01 molecules

**Figure 1:**
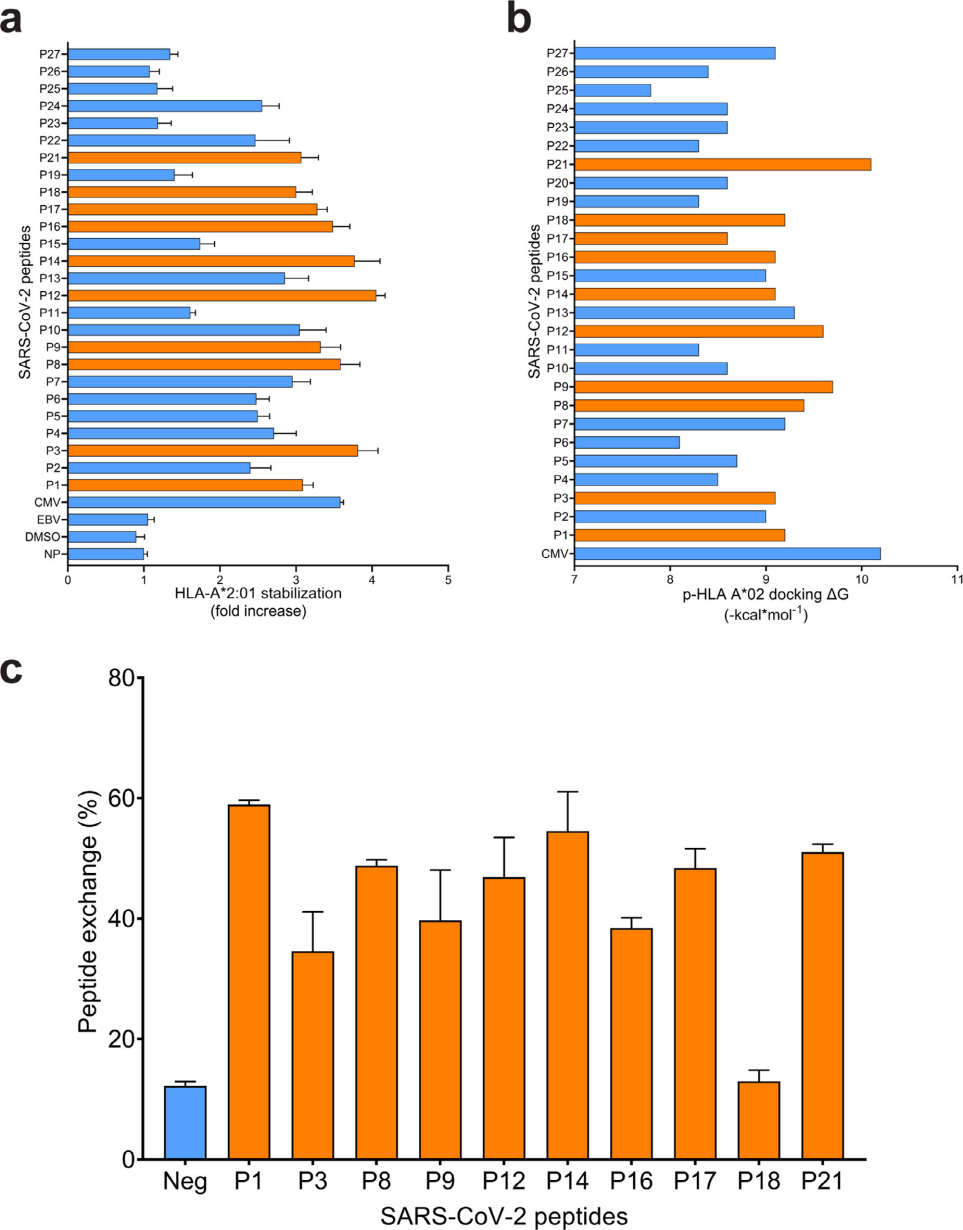
Stabilization of HLA-A*02:01 molecules by the *in silico* predicted SARS-CoV-2 peptides. (**a**) T2 cells were incubated for 16 hours either with each of the SARS-CoV-2 peptides (P1-P27, 100µM) individually, or with CMV pp65^495-503^ (as positive control), EBV EBNA3B^416-424^ (as negative control), DMSO (0.5%) or no peptide (NP). Then, the cells were stained with anti-HLA-A*02-APC antibody and analysed by flow cytometry. The median fluorescence intensities (MFIs) were taken as directly proportional to the levels of stabilized HLA*02*01 molecules. All results were normalized to the NP control and represented as fold increase to NP. Orange-colour bars denote peptides showing higher HLA*02*01 stabilization. Mean values of three independent experiments are shown. One-way ANOVA was used, p < 0.0001. (**b**) Free-energy increments of the molecular docking between each of the peptides and HLA-A*02:01. The DockTope and HLA-Arena algorithms were used. The binding energy (ΔG) of peptide-HLA-A*02:01 complexes was calculated using the PRODIGY server. Bars coloured as in (a). (**c**) Peptide exchange efficiency for HLA-A*02:01 was measured using a sandwich ELISA. The ten peptides showing higher HLA-A*02:01 stabilization were analysed. The EBV EBNA3B^416-424^ served as negative control (Neg). Shown are percentages of the CMV pp65^495-503^ positive control, which was set to 100. Represented are mean values of three independent experiments (one-way ANOVA p< 0.0001.

We used the T2 HLA-A2 shift assay to analyse the 27 peptides selected as putative HLA-A2-restricted epitopes. T2 is a lymphoma-derived cell line expressing HLA-A*02:01, but deficient in the transporter associated with antigen processing (TAP). These cells can only present exogenously added peptides. In the absence of peptide, the expression of HLA-A*02:01 on the surface of T2 cells is low, whereas the HLA-A*02:01/peptide complexes are stable and can be detected by immunofluorescence (T2-binding or shift assay) [38]. We evaluated by flow cytometry the HLA-A2 binding capacity of the 27 peptides on the basis of the mean fluorescence intensity (MFI) values obtained in three independent experiments compared with those of negative controls, which were T2 cells incubated under similar conditions with no-peptide (NP), DMSO or a non-HLA-A*02:01 EBV peptide. With the exception of P23 (E), P25 and P26 (both M), all other peptides displayed MFI values significantly above the controls, albeit with diverse fold enhancement ranging from 1.3-fold (P27) to 4.2-fold (P12) (Fig 1a). P20 was excluded from the study due to its poor solubility causing erratic values. Peptides P3, P12, and P14 stabilized HLA-A*02:01 to a higher extent (∼3.5 fold) than the well-known HLA-A*02:01 cytomegalovirus (CMV) pp65 epitope, used as positive control. Out of the 26 peptides tested in this assay we selected 10 with the highest HLA-A*02:01-stabilization scores (P1, P3, P8, P9, P12, P14, P16, P17, P18, P21) for subsequent characterization.

An *in silico* HLA-A2-peptide docking study was conducted to further estimate the binding affinities of these peptides to HLA-A*02:01 (Fig 1b). Docking provides a prediction of the structure of the HLA-peptide complex by computational methods using a scoring function as a proxy for the free energy of interaction. Tridimensional peptide-HLA docking predictions are shown in the supplementary information (Fig S5). The docking results revealed binding energies to HLA-A*02:01 <-8 kcal/mol for all but one peptide (P25). The ten peptides with higher stabilizing activity by the T2 HLA-A2 shift assay had binding energies <-9 kcal/mol, being P21 comparable to the CMV positive control peptide (<-10 kcal/mol) (Fig. 1b).

The affinity of CD8 T cell epitopes to MHC I can also be quantified by measuring peptide exchange. We used an ultraviolet-based peptide exchange system to measure the exchange efficiency of the above 10 peptides in comparison with a highly efficient HLA-A*02:01 binder peptide (CMV pp65), as positive control, and with the EBV EBNA3B 416-424 peptide, a non-HLA-A*02:01 peptide, as negative control (Fig 1c). Compared with CMV pp65, the exchange efficiencies of the peptides tested ranged from about 35% (P3) to nearly 60% (P1). P18 exhibited no significant exchange above the EBV negative control peptide, which was in contrast with the ability of P18 to stabilize HLA-A2 on T2 cells (Fig 1a) and to activate CD8 T cells (see below).

### 3.3 Unexposed donors show CD8 T cells reactive to SARS-CoV-2-derived peptides

Although previous studies have demonstrated cross-reactive CD4+ memory T cells in SARS-CoV-2 unexposed individuals, little is known about cross-reactive CD8 T cells [22,39]. To gain insight into the latter, we analysed whether and to which extent circulating CD8 T cells of unexposed donors specifically bind HLA*02:01 tetramers loaded separately with the 10 peptides selected in the previous section.

**Figure 2:**
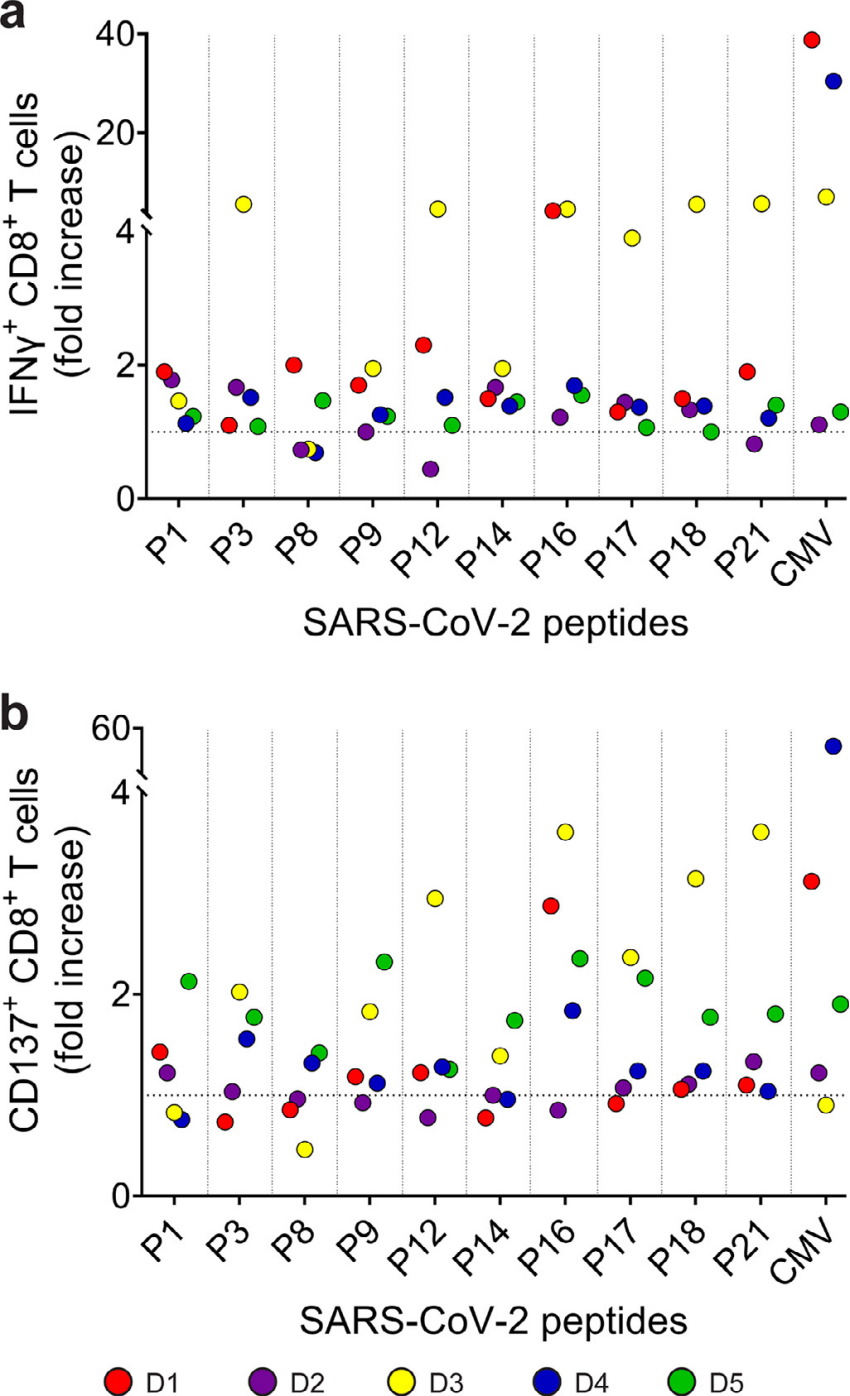
**Activation of CD8 T cells of unexposed donors upon stimulation with SARS-CoV-2 peptides.** The mDCs were loaded with the indicated SARS-CoV-2 peptides (10 µM) and co-incubated with autologous PBMCs for one week. (**a**) The mDC-PBMC co-culture was re-stimulated with the respective peptides for three hours, processed for IFN-γ secretion using an antibody catch assay and analysed by flow cytometry. As negative controls we ran samples with either No-Peptide (mean IFN-γ^+^=0.051%) or just Vehicle (DMSO at 0.05%, the same concentration than that added to the samples with peptide) (mean IFNγ^+^=0.10%). As positive control, we used the well-known CMV pp65^495-503^ peptide (10 µM). (**b**) After 24 h of re-stimulation with the respective peptide, the PBMCs were retrieved and analysed for CD137 expression. The data were normalized as fold increase over the Vehicle control (the dotted line denotes the background level measured in the DMSO control). Colours correspond to the indicated donors.

Peripheral blood mononuclear cells (PBMCs) of five donors were co-cultured for seven days with differentiated mature dendritic cells (mDCs) derived from autologous monocytes, which had been previously loaded with the respective peptides. SARS-CoV-2-specific CD8 T cells were detected by two surrogate activation markers: IFN-γ secretion and surface expression of CD137 (4-1BB), a member of the TNFR family with costimulatory function. As shown in Fig 2, all 10 peptides induced significant levels of IFN-γ secretion and CD137 expression in CD8 T cells, albeit in a donor-dependent manner. Donors D1, D3 and D5 showed higher frequencies of activated CD8 T cells (IFN-γ/CD137). Peptide P16 elicited the strongest activation, except in D2. Donor D3 exhibited the highest IFN-γ and CD137 frequencies for most peptides, yet showed unresponsive to P1 and P8 (Fig 2). Overall, for most peptides and donors, there were minor responses. Variability among donors was also observed for the CMV pp65 peptide that was used as control, which was in the range that could be expected from the CMV seroprevalence in Germany (∼56%) [40].

### 3.4 Ex vivo assessment of circulating memory CD8 T cells reactive to SARS-CoV-2-derived peptides in unexposed subjects

We analysed the frequencies of CD8 T cells reactive to SARS-CoV-2-derived peptides in PBMCs of six unexposed donors, distinguishing between naïve, memory and effector cells. For detection of specific CD8 T cells we used p-HLA-A*02:01 tetramer complexes of the peptides P3 (S-protein), P12 (pp1ab), P16 (pp1ab) and P21 (3a), which had high combined scores for activation and T2-binding. In addition, to distinguish between naïve, memory and effector T cells, we measured the surface expression of CD45RA, CD45RO and CCR7 in tetramer-binding/CD8 T cells. HLA-A*02:01 tetramers loaded with the Ins 10-18 peptide served as control for unspecific tetramer binding (Fig S3). To compensate for unspecific binding, we subtracted the tetramer-Ins 10-18 events in the naïve and memory subpopulations from the respective positive events for SARS-CoV-2-tetramers.

**Figure 3:**
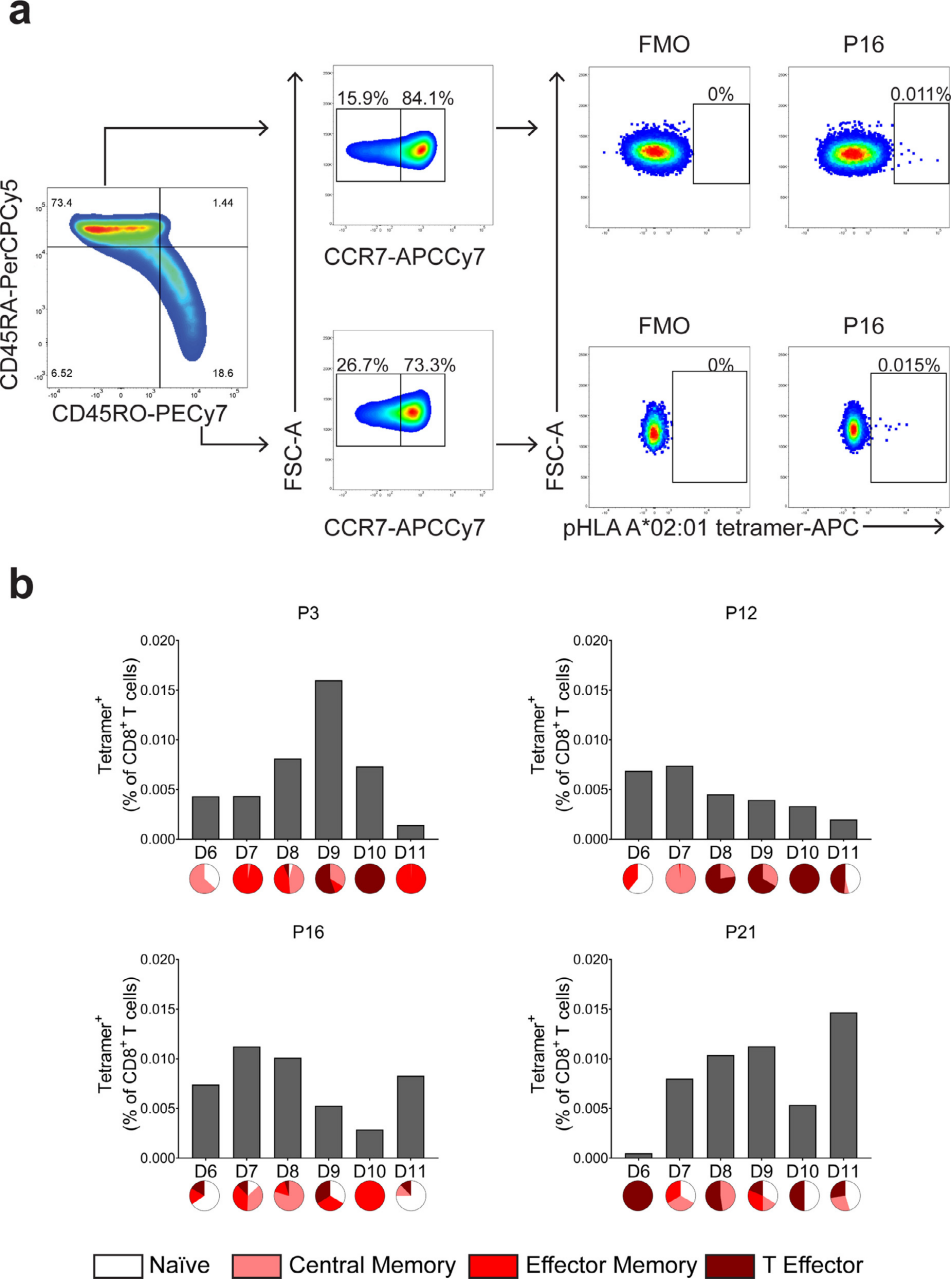
Naïve and memory CD8 T cells of unexposed donors bind p-HLA-A*02:01 tetramers bearing SARS-CoV-2 peptides. CD8 T cells were isolated from the PBMCs of five healthy unexposed donors and immediately after stained with CD3, CD8, CD45RA, CD45RO, CCR7 antibodies and with p-HLA-A*02:01 tetramers loaded with either P3, P12, P16, P21 or an irrelevant peptide (ins 10-18). (**a**) CD3**^+^**CD8**^+^** T cells gated into four quadrants based on anti-CD45RO-PECy7 and anti-CD45RA-PerCPCy5 staining. Then, the CD45RA**^+^**CD45RO**^−^** and CD45RA**^−^**CD45RO**^+^** cells were gated separately for p-HLA-A*02:01-APC tetramer positive cells due to significant differences in background levels. To avoid artefactual positive events a FMO control was made by skipping the pHLA A*02:01-tetramer staining. Finally, four subpopulations (naïve, central memory, effector memory and T effectors) were defined based on CCR7 expression. Representative results are shown comparing FMO and P16 loaded tetramer staining; insulin-tetramer positive events were subtracted from the respective memory subpopulations to compensate for unspecific background. Of 10^5^ events acquired per sample, about 90% were CD3**^+^**CD8**^+^** T cells. (**b**) The histograms show the percentages of p-HLA-A*02:01-tetramer positive CD8 T cells for six unexposed donors and the proportion of the four distinct memory subpopulations for each peptide and donor (pie charts at the bottom).

The Fig 3a shows the gating strategy used to identify SARS-CoV-2-reactive naïve and memory CD8 T cells. The frequencies of tetramer+/CD8 T cells in six unexposed individuals are represented in Fig 3b. All donors presented significant frequencies of tetramer-binding CD8 T cells against each of the four peptides, albeit to different extent. The P12-tetramer showed lower values in all donors. The highest values corresponded to the P3- and P21-carrying tetramers for donors D9 and D11, respectively.

Generally, the SARS-CoV-2-tetramer-positive cells were found mostly in the memory compartment. For the P3-tetramer, only donor D6 displayed mainly naïve cells, while in the others the tetramer-positive cells were predominantly memory T cells. In contrast, for P12 two subjects (D6 and D11) showed an increased proportion of naïve cells, while three others (D8, D9, D10) displayed T effector-oriented responses. A similar response profile was observed with P16, for which the T effector compartment was displaced by central/effector memory T cells in donors D7 to D10. Finally, it was not possible to detect tetramer-positive T cells in donor D6 with tetramers loaded with P21, and the other donors displayed a heterogeneous profile predominantly of the antigenically experienced compartment (Fig 3b).

### 3.5 **Long synthetic peptides induce expansion of SARS-CoV-2**-**reactive CD8 T cells**

**Figure 4:**
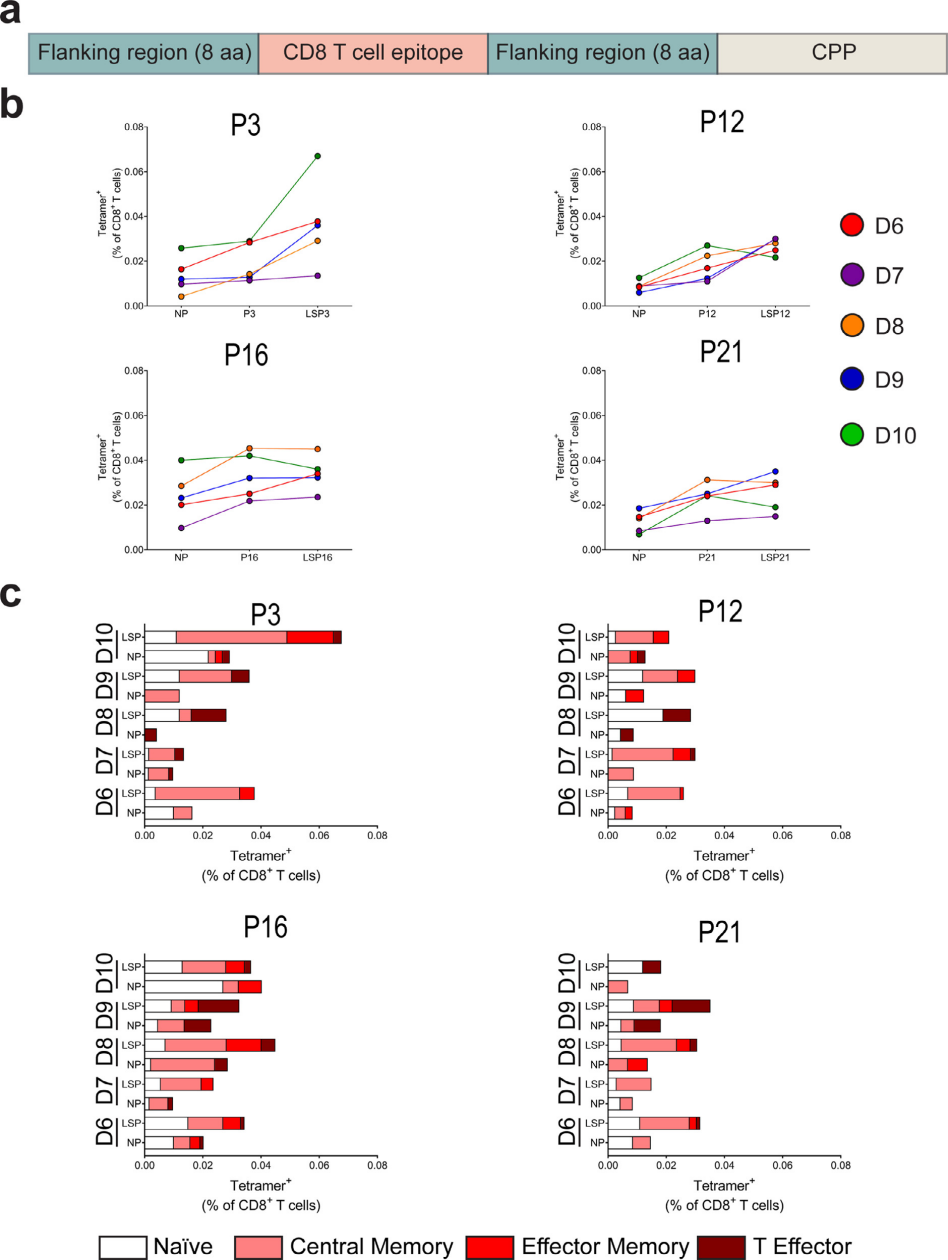
Long synthetic peptides containing SARS-CoV-2 peptides induced expansion of pHLA A*02:01 tetramer+ CD8 T cells in PBMCs of unexposed donors. (**a**) LSPs were designed to contain a T cell epitope sequence as in the native protein surrounded by short sequences (8 amino acids) and linked to a cell penetrating peptide (nine-arginine motif) at the C-terminus to facilitate cell entry. (**b**) mDCs from five unexposed donors were incubated for 3 hours with the indicated short (SP) or long synthetic peptides (LSP) or no peptide (NP). Then, co-cultures with autologous PBMCs (10:1 PBMCs:mDCs) was started and maintained for one week. Then, the PBMCs were processed for p-HLA-A*02:01 tetramer binding and the memory phenotype analysed with anti-CD45RA, -CD45RO and -CCR7 antibodies. The graphs show the percentages of CD8 T cells bound to p-HLA-A*02:01 tetramers (p= P3, P12, P16 or P21) for each donor. (**c**) Histograms representing the distribution of naïve and memory cells amid the p-HLA-A*02:01 tetramer-positive CD8 T cells in PBMCs of five unexposed donors. The PBMCs were co-cultured (at 10:1 ratio) with mDCs pre-incubated with the indicated peptides (LSPs) or no peptide (NP). The fractions of naïve, central memory, effector memory and T effector cells are displayed in different colours within the columns. To compensate for unspecific tetramer binding, we used an HLA-A*02:01 tetramer bearing the insulin epitope HLVEALYLV (Ins 10-18) as control and the background levels obtained with it were subtracted from the main samples.

Long synthetic peptides (LSPs) have been extensively used as vaccines, as they facilitate and enhance the presentation of putative epitopes by dendritic cells to autologous CD8 T cells [41,42]. Typically, LSPs are 25-35 amino acids long, carrying a CD8 T cell epitope of the target protein [43]. To determine whether predicted peptide epitopes of SARS-CoV-2 are naturally processed and capable of eliciting a functional T cell response, we designed four LSPs each carrying one of the peptides P3, P12, P16 or P21 with flanking amino acid sequences as in the natural viral proteins (Fig 4a). In addition, a C-terminal polyarginine sequence (R9) was added to each peptide to facilitate penetration of the LSP into the cells [44,45]. The sequences of the LSPs are available in the supplementary information (Table S6).

We loaded mDCs isolated from five healthy unexposed donors either with short peptide (P), LSP or no peptide as control and started a co-culture with autologous PBMCs for seven days. Finally, we measured the expansion of peptide-specific CD8 T cells by using p-HLA-A*02:01-tetramers (p= P3, P12, P16 or P21) after subtracting background levels obtained with the insulin tetramer and distinguishing naïve, memory and effector subpopulations within the tetramer+/CD8 T cells using antibodies against CD45RA, CD45RO and CCR7. Representative results of the tetramer staining with the ins 10-18 tetramer and the P3 tetramers with or without LSP3 stimulation are shown in the supplementary material (Fig S7).

As expected, all donors exhibited basal frequencies of tetramer-binding CD8 T cells (Fig 4b, NP). The short peptides induced an expansion of the CD8 T cell populations binding the corresponding tetramers. Likewise, LSPs carrying these epitopes evoked an increase in frequency of tetramer-binding CD8 T cell that, for most donors, was comparable or higher than that seen with the respective short peptides, and only in a few cases were similar or slightly lower (Fig 4b). As could be expected, the frequencies of CD8 T cells binding tetramer were significantly higher than those measured in PBMCs not co-cultured with mDCs. The analysis of the different memory subsets showed that, in general, there was an expansion of T cells with central and effector memory phenotype and a slight increase of the naïve subset (Fig 4c). Overall, these results suggested that the predicted epitopes contained in the LSPs were indeed true HLA-A*02:01-restricted epitopes, as they were efficiently processed and presented by the mDCs after intracellular delivery.

### 3.6 TCR clonotypes reactive to SARS-CoV-2 are present comparably in unexposed donors and COVID-19 recovered patients

We then investigated the frequencies of TCR clonotypes reacting to SARS-CoV-2 peptides in unexposed donors and COVID-19 convalescent patients. We used the ImmuneCODE™ database (Adaptive Biotechnologies), which was created by a number of individuals and organizations and made freely available [32] to identify TCR clonotypes associated with SARS-CoV-2 and to follow the dynamics of the T cell repertoire in the course of infection. This database contains information on *TCRβ* sequences and their respective frequencies among over 1,400 subjects exposed to SARS-CoV-2 or that were suffering COVID-19 or had recovered from it. In addition, we used a high throughput multiplex tool (Multiplex Identification of Antigen-Specific T-Cell Receptors Assay: MIRA) that enables the identification of epitope-specific TCRs to large numbers of SARS-CoV-2 antigens simultaneously by combining stimulation of PBMCs from donors with pools of viral peptides with cell sorting based on upregulation of activation markers (CD137) and TCR sequencing of SARS-CoV-2 exposed subjects and naïve unexposed donors. The MIRA dataset contains sequences of more than 135,000 high-confidence SARS-CoV-2-specific TCRs [32].

**Figure 5:**
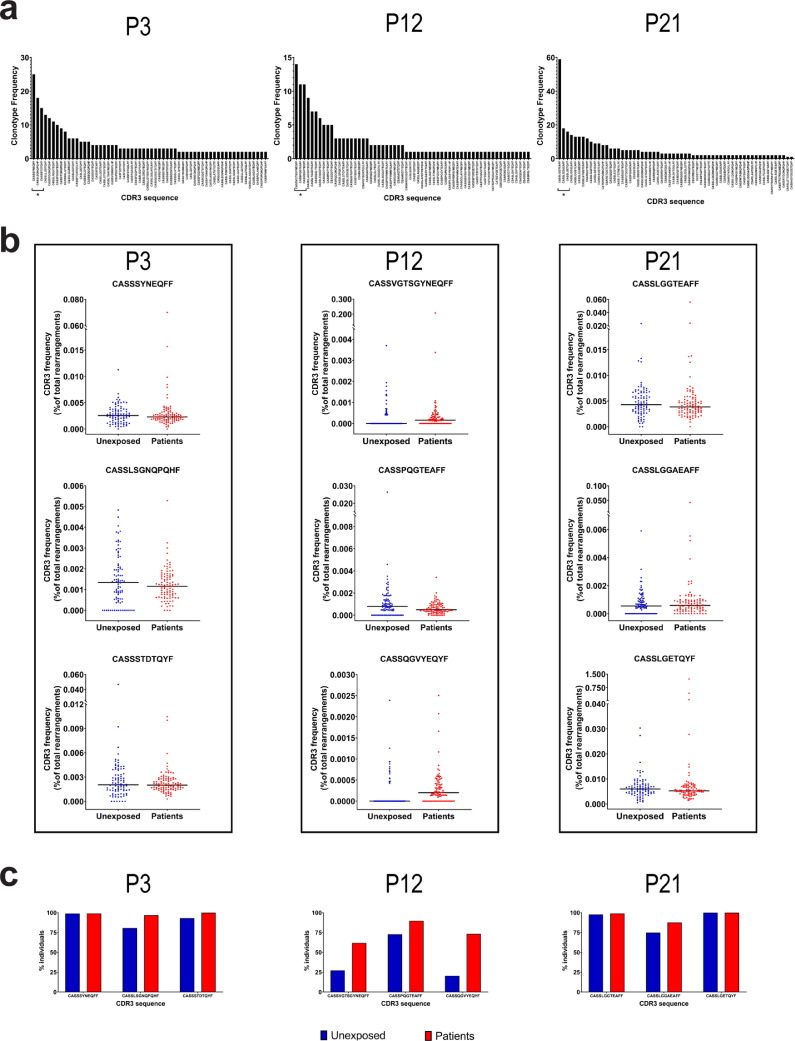
**Analysis of SARS-CoV-2-reactive T cell clonotypes in unexposed donors and in COVID-19 recovered individuals.** (**a**) Histograms showing the most frequent T cell CDR3 sequences (clonotypes) activated by pulsing PBMCs of COVID-19 patients (HLA A*02:01+) with one of the following peptide-pools: (i) P3: SIIAYTMSL (P3), ASQSIIAYTM, RSVASQSII, SQSIIAYTM and VASQSIIAY; (ii) P12: TMADLVYAL (P12) and YTMADLVYA; and (iii) P21: LLYDANYFL (P21), LLYDANYFLC, LYDANYFLCW, NPLLYDANY, PLLYDANYFL and YDANYFLCW. In this analysis, the MIRA immunosequencing results of 31 HLA A*02:01+ COVID-19 patients were considered. (**b**) Comparison of the frequencies of P3, P12 and P21 pool-specific clonotypes in PBMCs of COVID-19 patients and unexposed donors. The three most represented clonotypes identified with each peptide pool in COVID-19 patients were analysed for their frequencies in the repertoire of COVID-19 recovered patients (97 individuals) and healthy unexposed donors (88 individuals). The values correspond to the median frequencies of the clonotypes displayed at the top of each graph. A Mann-Whitney-Wilcoxon test was performed to compare clonotype frequencies among the two groups, p values were as follows: P3-CASSSYNEQFF p=0.480; P3-CASSLSGNQPQHF p=0.534; P3-CASSSTDTQYF p=0.943; P12-CASSVGTSGYNEQFF p=0.0027; P12-CASSPQGTEAFF p=0.00438; P12-CASSQGVYEQYF p<0.0001; P21-CASSLGGTEAFF p=0.299; P21-CASSLGGAEAFF p=0.399; P21-CASSLGETQYF p=0.285. (**c**) Percentages of patients and unexposed individuals showing at least one clonotype with the respective CDR3 sequence. All data analyses were performed using the ImmunoSEQ Analyzer 3.0 of Adaptive Biotechnologies.

We used the ImmuneCODE™ and MIRA datasets to compare the TCR sequences of CD8 T cells reactive to SARS-CoV-2. First, we retrieved in the MIRA dataset the frequencies of the SARS-CoV-2-specific clonotypes of HLA-A*02:01 COVID-19 patients (31 individuals) searching exclusively those reacting to peptide pools including either P3, P12 or P21, used herewith to designate the respective peptide pools. Peptide P16 was not found in any of the peptide pools used to obtain the MIRA dataset, hence it was not included in the analysis. We found numerous shared clonotypes reactive to each peptide pool (Fig 5a). For the P3 pool, the most prevalent CDR3 was CASSSYNEQFF, which was identified 25 times in 14 of the 31 patients. The most prevalent CDR3 sequence (CDR3-s) for the P12 pool was CASSVGTSGYNEQFF identified 14 times in 11 patients. Finally, for the P21 pool the most prevalent CDR3 was CASSLGGTEAFF, detected 59 times in 18 patients (Fig 5a). Clonotypes frequencies found in the MIRA database patients are displayed in the supplementary information (Table S8 and S9).

For further analyses we focused on the three most prevalent CDR3 clonotypes identified for each peptide pool. Thus, we retrieved from the MIRA database the frequencies of the three CDR3-s in the *TCRβ* repertoire of patients recovered from COVID (97 individuals) and compared them with the frequencies in the unexposed control group (88 individuals), as shown in Fig 5b. In the P3 and P21-associated CDR3 sequences we found a tendency for higher median frequencies in the unexposed donors compared to the recovered patients group. However, the differences observed proved not to be statistically significant (*p* ≥ 0.05 by the Mann–Whitney–Wilcoxon test). In the P12, the CDR3 CASSVGTSGYNEQFF and CASSQGVYEQYF were more frequent in the recovered donors, while CASSPQGTEAFF was more represented in the unexposed group (Fig 5b). The differences observed for these three clonotypes were statistically significant (p value < 0.05). Furthermore, besides the CDR3 clonotype frequencies, we also calculated the percentage of individuals of each group (unexposed donors and recovered patients) displaying at least once these clonotypes. This analysis revealed that the proportion of those individuals was higher in the group of recovered patients, especially in the case of P12 (Fig 5c). These results are consistent with our data showing that SARS-CoV-2-reactive T cell clonotypes are present in unexposed individuals.

### 3.7 SARS-CoV-2 peptides share homology with previous human coronaviruses

Cross-reactive, pre-existing T cell responses to common coronavirus might explain the high prevalence of SARS-CoV-2 reactive CD8 T cells among unexposed individuals and could have a significant impact on the immune reactivity to SARS-CoV-2 infection. We analysed the homology of the ten peptides we selected with HLA A*02:01 restricted T cell epitopes from other human coronaviruses. We used the NetCTLpan T cell epitope prediction tool from IEDB to identify HLA A*02:01-restricted T cell epitopes from the complete viral proteome of each of the studied coronaviruses. Then we aligned the resulting predicted epitopes with their respective homolog from SARS-CoV-2. For this comparison we took into consideration only coronaviruses that in the past have proven to be able to infect humans.

We found that P12, P16 and P18 showed relatively high similarity among several human coronaviruses. On the other hand, P1, P3, P8, P9, P14, P17 and P21 share homology with SARS-CoV and MERS-CoV (Table II).

Additionally, a pairwise sequence alignment was carried out between the peptides and the proteins of related coronaviruses including SARS-CoV, MERS-CoV, OC43, HKU1, NL-63, and 229E (Fig S10). As expected, the highest degree of homology was with SARS-CoV (100% P12, P14 and P17; 96% P3 and P21; 93% P18 and 81% P1, P8 and P9). However, the degree of similarity of SARS-CoV-2 epitopes to OC43, HKU1, NL-63, and 229E was much lower. Although cross reactivity between different coronaviruses in the context of HLA-A*02:01 restricted T cell epitopes is theoretically possible, the outbreaks of SARS-CoV and MERS-CoV infections were endemic and, therefore, do not help understand the high frequency of SARS-CoV-2 reactive CD8 T cells in healthy unexposed donors.

## 4 Discussion

The development of optimal immunotherapies and vaccines to treat and prevent SARS-CoV-2 infections requires sufficient understanding of the specific T cell responses to the viral antigens. Yet the distinct mechanisms underlying the nature and degree of cellular immunity to SARS-CoV-2 remain elusive. For instance, the strong T cell reactivity seen in COVID-19 patients suffering severe disease seems to be associated with a dysfunctional control of the infection by the immune system, which can be life threatening. Previous studies have shown broad T cell responses in a majority of subjects recovering from COVID-19, in particular a high frequency of CD4+ T cells specific to the spike protein [13,23]. Furthermore, patients suffering mild disease have higher frequencies of SARS-CoV-2-specific CD8 T cells compared with those with severe symptoms, implying a protective effect, while a pathogenic role of CD4+ T cells has been suggested in severe disease [24,46].

Most current vaccines target the SARS-CoV-2 spike protein to induce neutralizing antibodies and T cell responses. However, the extent of protection and its duration remains uncertain, especially considering the emergence of new virus mutants that generate variants of concern against which the antibody responses triggered by current vaccines could not protect from severe progression implicating the importance of T cell responses. Therefore, there is a need for elucidating the T cell reactivity to SARS-CoV-2. In this study we have investigated the frequency, specificity and distribution of pre-existing SARS-CoV-2 cross-reactive CD8 T cells in healthy unexposed donors. Using the NetCTLpan T cell epitope prediction tool (IEDB) we analysed the entire SARS-CoV-2 proteome and selected 27 putative HLA-A*02:01-restricted peptides on the basis of their high combined scores for proteasomal cleavage, TAP transport and MHC class I binding capability (Table I). When tested by the T2 HLA-A*02:01 shift assay the majority of these peptides, except P25 and P26, showed significant stabilization values above the negative controls. The molecular docking analysis also revealed relevant binding energies <-8 kcal.mol^−1^ for all peptides except P25 (Fig. 1b). A peptide-exchange assay confirmed the ability of these peptides to bind HLA-A*02:01. Intriguingly, one peptide (P18) showed no significant exchange above the EBV negative control, which is in contrast with the ability of P18 to bind and stabilize HLA-A*02:01 on T2 cells (Fig 1a and b) and activate CD8 T cells in some donors (Fig 2a and b). Loaded onto mDCs and co-cultivated with autologous CD8 T cells of SARS-CoV-2 unexposed subjects these peptides induced activation in a significant subset of cells (IFN-γ secretion or overexpression of CD137). Interestingly, we observed inter- and intra-donor variability in the frequencies of activated CD8 T cells with the different peptides. Overall, donor D3 showed the highest frequencies, followed by D5 and D1, while D2 showed only a mild activation (IFN-γ) with P1, and donor D4 reacted mildly to P3 and P16, but strongly to the CMV peptide. In good correlation with the HLA-A*02:01 stabilization and docking scores, the P16 peptide (pp1ab-derived) induced higher frequencies of CD137 overexpressing cells except with donor D2. In contrast, P1, P8, P9, and P14 had a high stabilizing activity and binding affinity to HLA-A*02:01 (Fig. 1), yet they evoked lower T cell activation than expected. However, such discrepancies between different assays with T cells have been documented previously [25,46] and may be related to differences in sensitivity and functional relevance of the assays.

Based on their CD8 T cell activation capacity we chose four representative peptides (P3, P12, P16 and P21) for further analysis using tetramers (Fig. 3). Freshly isolated PBMCs of six SARS-CoV-2 unexposed donors showed mostly memory tetramer-binding CD8 T cells, albeit with variable frequencies for the different peptides. We detected naïve and effector T cells, in particular for P12 and P21. No recognizable pattern was associated with any peptide between the different donors and, conversely, each donor showed different subpopulation frequencies for each peptide. Nevertheless, all donors showed central and effector memory cells binding tetramers, except D1 with P21 and D5 with P3, P12 and P21. Furthermore, tests with LSPs demonstrated that the four peptides are indeed HLA-A*02:01 epitopes able to elicit higher frequencies of tetramer-binding CD8 T cells compared to the respective short peptides (Fig. 4b). As expected from the previous results with fresh non-cultivated PBMCs, the expanded cell subsets in these assays exhibited naïve and central/effector memory phenotypes. Recently, it was found that the majority of SARS-CoV-2-specific CD8 T cells from COVID-19 convalescent individuals display central and effector memory phenotype [46]. Taken together, these data are suggestive of cross-reactive pre-existing CD8 T cells which, in view of the lack of homology with common cold coronaviruses cannot be explained from previous contact with them (Table 2 and Fig. S10).

**Table 2:** Human coronaviruses epitope comparison in the context of HLA A*02:01. The studied peptides from SARS-CoV-2 where compared in similarity to their counterparts in the rest of human infecting coronaviruses. Asterisks indicate matching amino acids with the respective SARS-CoV-2 epitope. The numbers indicate the position of the epitope in the viral proteins.

Coronavirus	**P1**	**P3**	**P8**	**P9**	**P12**	**P14**	**P16**	**P17**	**P18**	**P21**
	Spike protein		Replicase 1ab					Nucleoprotein		ORF3a
**SARS-CoV-2**	YLQPRTFLL 269-277	SIIAYTMSL 691-699	YLATALLTL 1675-1683	VMVELVAEL 84-92	TMADLVYAL 4515-4523	LLADKFPVL 6246-6254	FLIGCNYL 7003-7010	LLLDRLNQL 222-230	YLGTGPEAGL 112-121	LLYDANYFL 139-147
**SARS-CoV**	**K*T**M* 256-264	**V****** 673-681	**SSV**A* 1652-1660	KV******M 84-92	********* 4492-4500	********* 6223-6231	****A*** 6980-6987	********* 223-231	********S* 113-122	********V 139-147
**MERS-CoV**	K***L**** 317-325	MEA***S** 943-951	**NAVIM** 1593-2001	PR*Y**ER* 86-94	**M****** 4502-4510	***GS*DKV 6229-6237	**L*I*** 6980-6987	D**N**Q** 217-225	*T******A* 102-111	—
**HKU1**	—	—	—	—	**L**C*** 4589-4596	—	****I*** 7096-7103	—	******Y*NA 126-135	—
**NL-63**	—	—	—	—	**M*****M 4155-4163	—	****I*** 6628-6635	—	******HKD* 80-89	—
**229E**	—	—	—	—	**M**CF** 4185-8193	—	*VV*I*** 6662-6669	—	******HKD* 82-91	—
**OC43**	—	—	—	—	**L**C*** 4492-4500	—	****I*** 7001-7008	—	—	—

A careful analysis of the ImmuneCODE™ and MIRA datasets revealed relatively large numbers of frequent shared clonotypes reactive to discrete peptide pools containing P3, P12 or P21 among the HLA-A*02:01 COVID patients (Fig. 5a). Interestingly, the same clonotypes were also present in unexposed individuals, as illustrated in fig. 5b for the three most prevalent clonotypes. These findings are in agreement with previous reports showing SARS-CoV-2 reactive T cells in a high proportion of unexposed individuals [11,13,15]. One would expect these shared clonotypes to be more prevalent in patients. In contrast, a comparison of the frequencies of the reactive clonotypes in the repertoire of unexposed and COVID19 recovered patients revealed that the median frequencies of these clonotypes were comparable in patients and unexposed subjects, with only slight differences, as illustrated in Fig. 5b for the three most represented clonotypes reactive to each peptide pool. These data diverge from previous reports in which significantly increased CD8 T cell responses were described in COVID-19 recovered patients compared with unexposed individuals [13,46,47]. A possible explanation might be that our analysis focused on clonotypes shared between many different patients, likely biasing the search to SARS-CoV-2 cross-reactive public clonotypes. In this context, it is worth to note a recently published meta-analysis of 18 studies on 852 individuals recovered from COVID -19, which allowed to identify a number of immunodominant epitopes in COVID-19 patients. Among these there were two HLA-A*02:01 epitopes of this study (P1 and P21) [48]. Nevertheless, the detection of “reported-as-reactive” clonotypes in the TCR repertoire of unexposed individuals represents a more sensitive approach, which could explain the high frequency of donors carrying reactive clonotypes. Moreover, for the CDR3 clonotypes analysed the percentage of individuals with detectable reactive clonotypes was higher in the dataset of recovered patients compared to the unexposed donors (Fig. 5c). Public T cell clonotypes have been described as highly prevalent clonotypes shared among different donors [[49], [50], [51]]. The fact that SARS-CoV-2-reactive clonotypes have comparable prevalence in convalescent and unexposed individuals might be due to public clonotypes being outperformed by private subject-specific T cell clonotypes with higher binding affinities, or to the response being biased to highly immune dominant epitopes.

In summary, we demonstrate the existence of naïve and memory SARS-CoV-2-reactive CD8 T cells in peripheral blood of unexposed healthy subjects. The homology shared by SARS-CoV-2 with seasonal coronaviruses can explain only in part the high frequency with which these cells are found in unexposed individuals. Further, the relatively high prevalence of naïve CD8 T cells involved supports that a significant proportion of these cells belongs to the public shared repertoire. Yet, the role of cross-reactive CD8 T cells during SARS-CoV-2 primary infection and their significance in unexposed individuals remain unclear. One possibility is that their TCRs are essentially prevalent in the public repertoire, either due to exposure to common viral or non-viral antigens or because they belong to a subset of highly cross-reactive clonotypes. To gain further insight into the cross-reactive repertoire we are currently using the epitopes characterized in this study to isolate specific paired TCR clonotypes among the ample, unbiased repertoire of unexposed donors, Additionally, these epitopes might be of prophylactic and therapeutic value against COVID-19 in the form of LSP-vaccines. Since T cell epitopes are well conserved among SARS-CoV-2 variants [48,52], our data support that a pan-coronavirus immunization is feasible if T cell target epitopes like those characterized here are included in next generation vaccines. Nevertheless, the presence of TCR clonotypes reactive to SARS-CoV-2 in unexposed individuals does not warrant the functionality of the T cells expressing them.

Limitations of our study are that it was based on a specific MHC I allele (HLA-A*02:01) and the low numbers of samples and epitopes tested. Further studies investigating other HLA alleles should determine whether cross-reactivity is a broad-spectrum phenomenon. Moreover, the identification of SARS-CoV-2-reactive paired TCR clonotypes should help understand the nature of such cross-reactivity.

## Contributors

IQF contributed to study design, conducted experimental work, data analysis, interpretation and presentation, and helped write the manuscript; MP performed *in silico* modelling and similarity analyses and helped write the manuscript; EF contributed in the *in silico* modelling and the flow cytometry data. ACA conceived the idea, designed the project, interpreted the results and wrote the manuscript. IQF and ACA verified the data. All authors had full access to all the data in the study and approved the final version of the manuscript.

## Data sharing

The raw data of this study are deposited in the database of the German Cancer Research Center (DKFZ), and can be provided to inquirers upon reasonable requests.

## Funding

See the Acknowledgements section.

## Declaration of Competing Interest

The authors declare no competing interests.

## Appendix Supplementary materials

Image, application 1

Image, application 2
