# Supplementary material for: Immunogenic T cell epitopes of SARS-CoV-2 are recognized by circulating memory and naïve CD8 T cells of unexposed individuals
Source: eBioMedicine. 2021 Oct 6;72:103610. doi: 10.1016/j.ebiom.2021.103610 (PMC8493415; doi:10.1016/j.ebiom.2021.103610)
Supplement: Supplementary file 1 [file mmc1.docx]

Caption for supplementary material

**Figure S1.** Dendritic cell differentiation from human peripheral blood monocytes. After the differentiation with IL-4 and GM-CSF, cells were retrieved and stained using an anti-CD11c-PE. Loosely adherent cells were retrieved and analyzed following the depicted gating.

**Figure S2.** Gating strategy for the quantification of IFNγ secretion and CD137 activation markers in CD8+ T cells stimulated with either vehicle control (DMSO 0.05%), CMVpp65 495-503 or P16 loaded dendritic cells. (**a**) Gating strategy to focus in CD8+ T cells. (**b**) An IFNγ catch assay was used to detect activated CD8+ T cells after three hours of peptide re-stimulation. (**c**) Quantification of CD8+ T cells with increased expression of CD137 activation marker after 24h of peptide re-stimulation. CMV pp65 (495-503) induced a strong activation in some of the donors and was used as a positive control. Representative data corresponds to a single donor.

**Figure S3**. Insulin 10-18 peptide loaded tetramer (HLA-A2*02:01) staining of CD8+ T cells. Six HLA-A*02:01 positive donors CD8+ T cells were stained with CD45RA, CD45RA, and CCR7 directed antibodies to distinguish naïve, memory and effector subpopulations. Bars depict the tetramer positive cells among the CD8+ T cells and the pie charts show the proportion of each memory phenotype within the tetramer+ cells.

**Figure S4**. pHLA A*02:01-tetramer staining validation. PBMCs from one HLA A*02:01 negative and three HLA A*02:01 positive donors were stained anti-CD8-FITC, anti-CD3-PEcy7, insulin 10-18 loaded tetramers-PE and SARS-CoV-2 peptides loaded tetramers-APC. CD8+ T cells were analyzed after tetramers co-staining. Gating of the positive events was defined by using a FMO for each channel (either PE or APC). No double positive events (insulin tetramer-PE+ / P3-21 tetramer-APC+) were detected in any of the analyzed samples. Results are shown for one HLA-A*02:01 negative donor (Donor A) and three HLA-A*02:01 positive donors (Donors B, C and D). The donors used for this validation experiment were different from the ones used throughout the article.

**Figure S4**. pHLA A*02:01-tetramer staining validation. PBMCs from one HLA A*02:01 negative and three HLA A*02:01 positive donors were stained anti-CD8-FITC, anti-CD3-PEcy7, insulin 10-18 loaded tetramers-PE and SARS-CoV-2 peptides loaded tetramers-APC. CD8+ T cells were analyzed after tetramers co-staining. Gating of the positive events was defined by using a FMO for each channel (either PE or APC). No double positive events (insulin tetramer-PE+ / P3-21 tetramer-APC+) were detected in any of the analyzed samples. Results are shown for one HLA-A*02:01 negative donor (Donor A) and three HLA-A*02:01 positive donors (Donors B, C and D). The donors used for this validation experiment were different from the ones used throughout the article.

**Table S6**. Long synthetic peptides sequences. Eight aminoacids from the native protein sequence were added to each flank of the predicted epitope (highlighted in red). A nine arginine tail was added in the C-terminal as a cell penetrating domain to facilitate cellular entry.

**Figure S7**. Representative figure for the gating of pHLA A*02:01 tetramer+ cells after LSP3 stimulation of CD8+ T cells. After a one week stimulation of PBMCs with either no peptide (NP) or LSP3 loaded dendritic cells, cells were retrieved for HLA A*02:01 tetramer APC staining. (**a**) To compensate for unspecific binding to tetramers, the PBMCs were stained using HLA A*02:01 tetramers loaded with insulin 10-18. (**b**) Representative results for the stimulated CD8+ T cells from donor 10 (D10) stained with the P3 HLA A*02:01 tetramers. The LSP3 stimulated cells show a 2.3 fold expansion in terms of P3 HLA A*02:01 tetramer + CD8+ T cells.

**Table S8**. Single CDR3 frequencies in the MIRA database (top 60 most frequent clones) for each of the peptide pools containing the selected peptides. Patients were filter based on HLA-A*02:01 expression, each time a CDR3 appeared was considered as a hit.

**Table S9**. Proportion of patients within the MIRA database with the following SARS-CoV-2 reactive T cell clonotypes. Proportions are shown for the top three most frequent reactive clonotypes for each of the peptide pools containing the P3, P12 or P21 peptides. 31 COVID-19 HLA-A*02:01+ patients were considered for this analysis.

**Figure S10**. Heatmap representation of a similarity matrix between SARS-CoV-2 epitopes and other human infecting coronaviruses. Each SARS-CoV-2 predicted epitope was separately aligned with each protein of other coronaviruses including SARS-CoV, MERS-CoV, OC43, HKU1, NL-63, and 229E. The similarity percentage between each pairs are indicated in numbers. Yellow and blue colors refer to the higher and lower similarity, respectively.
