# Supplementary material for: Immunogenic T cell epitopes of SARS-CoV-2 are recognized by circulating memory and naïve CD8 T cells of unexposed individuals
Source: eBioMedicine. 2021 Oct 6;72:103610. doi: 10.1016/j.ebiom.2021.103610 (PMC8493415; doi:10.1016/j.ebiom.2021.103610)
Supplement: Supplementary file 2 [file mmc2.docx]

**SUPPLEMENTARY FILES – Quiros et al.**


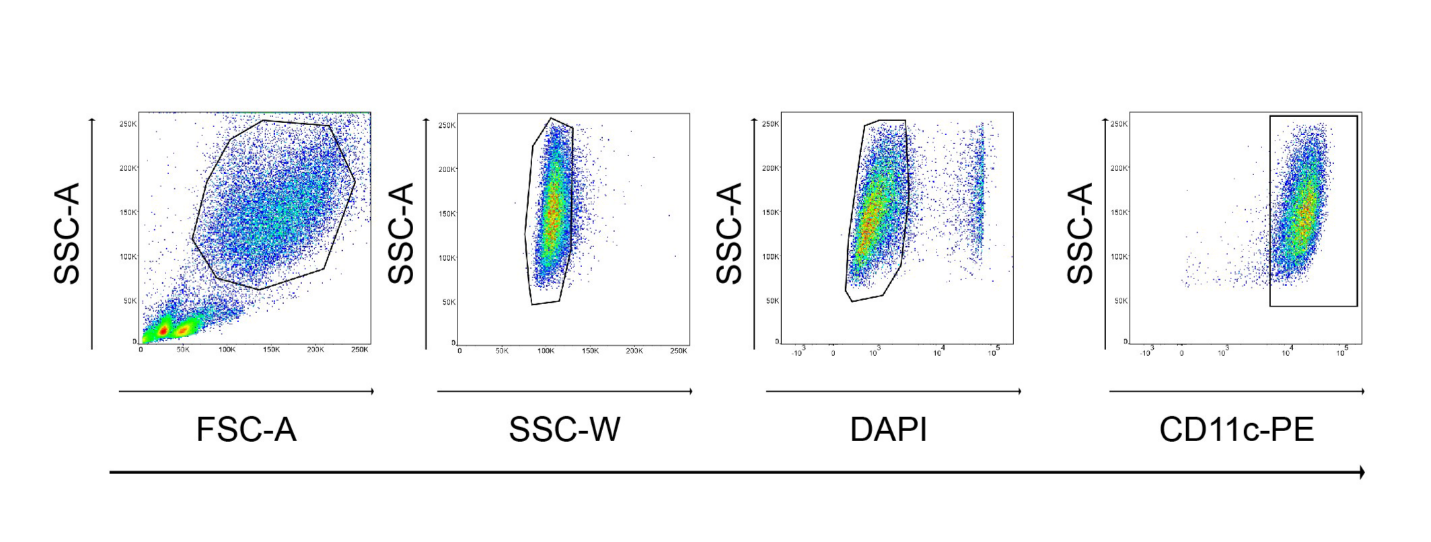


**Figure S1.** Dendritic cell differentiation from human peripheral blood monocytes. After the differentiation with IL-4 and GM-CSF, cells were retrieved and stained using an anti-CD11c-PE. Loosely adherent cells were retrieved and analyzed following the depicted gating.


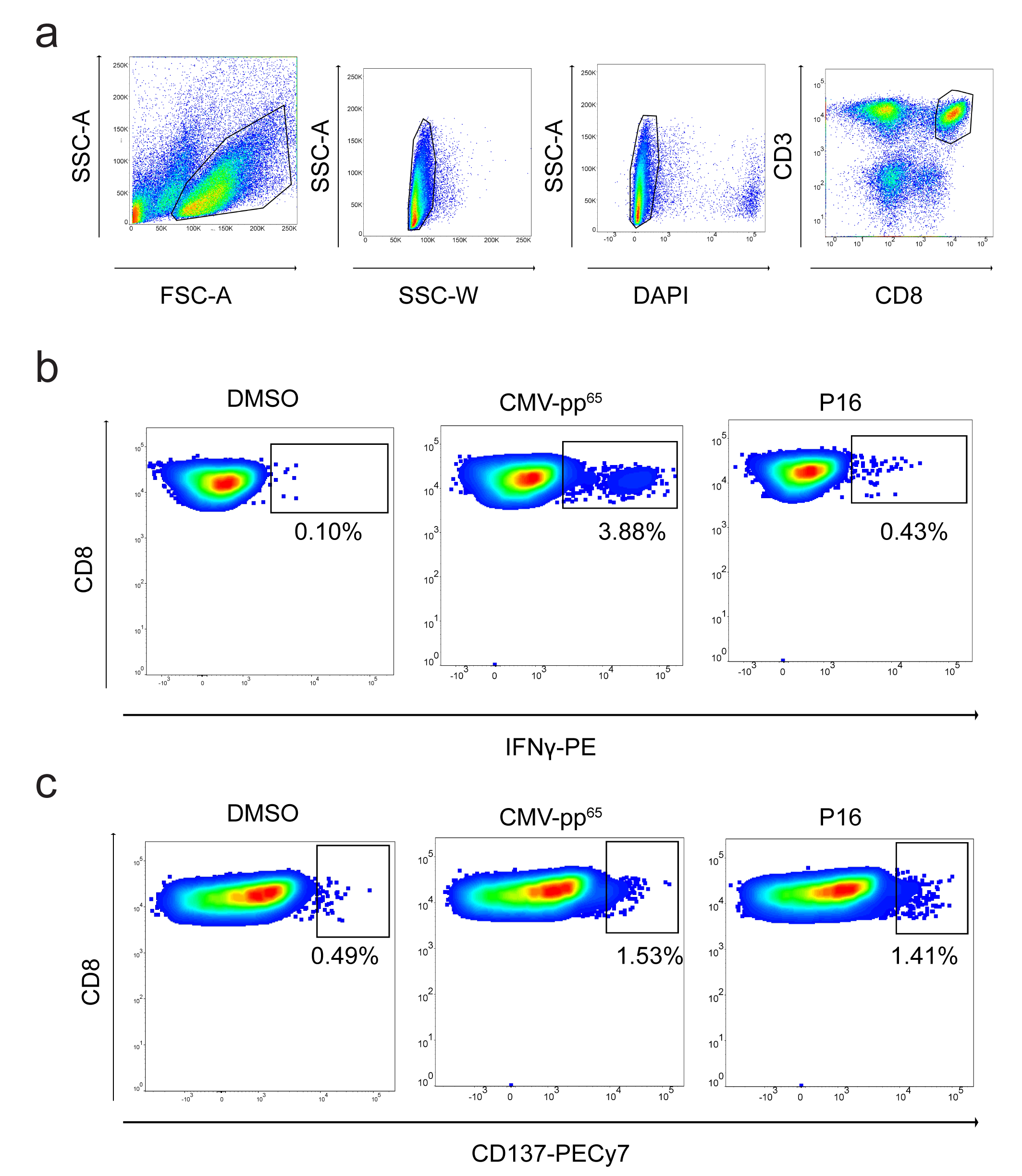


**Figure S2.** Gating strategy for the quantification of IFNγ secretion and CD137 activation markers in CD8+ T cells stimulated with either vehicle control (DMSO 0.05%), CMVpp65 495-503 or P16 loaded dendritic cells. (**a**) Gating strategy to focus in CD8+ T cells. (**b**) An IFNγ catch assay was used to detect activated CD8+ T cells after three hours of peptide re-stimulation. (**c**) Quantification of CD8+ T cells with increased expression of CD137 activation marker after 24h of peptide re-stimulation. CMV pp65 (495-503) induced a strong activation in some of the donors and was used as a positive control. Representative data corresponds to a single donor.


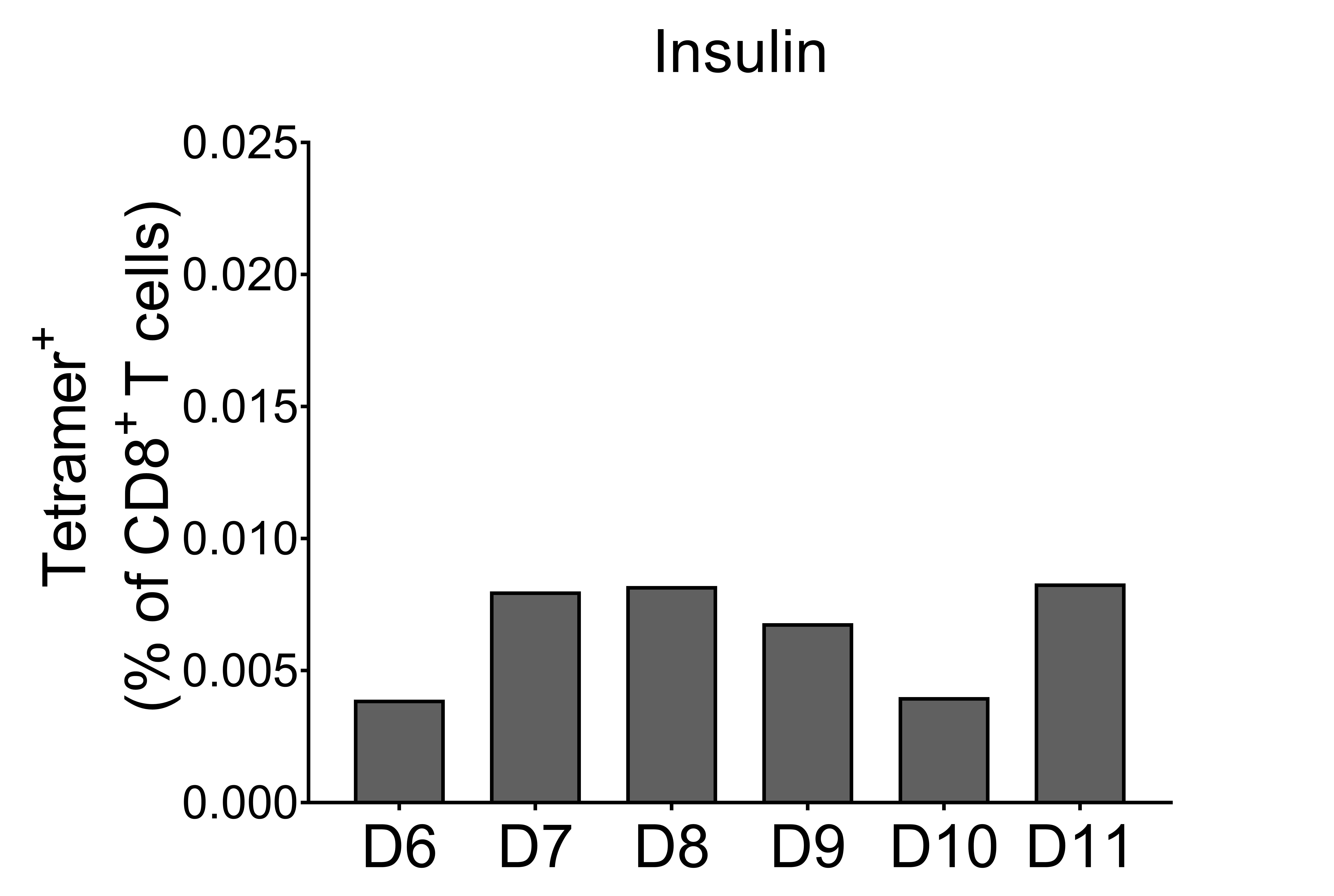

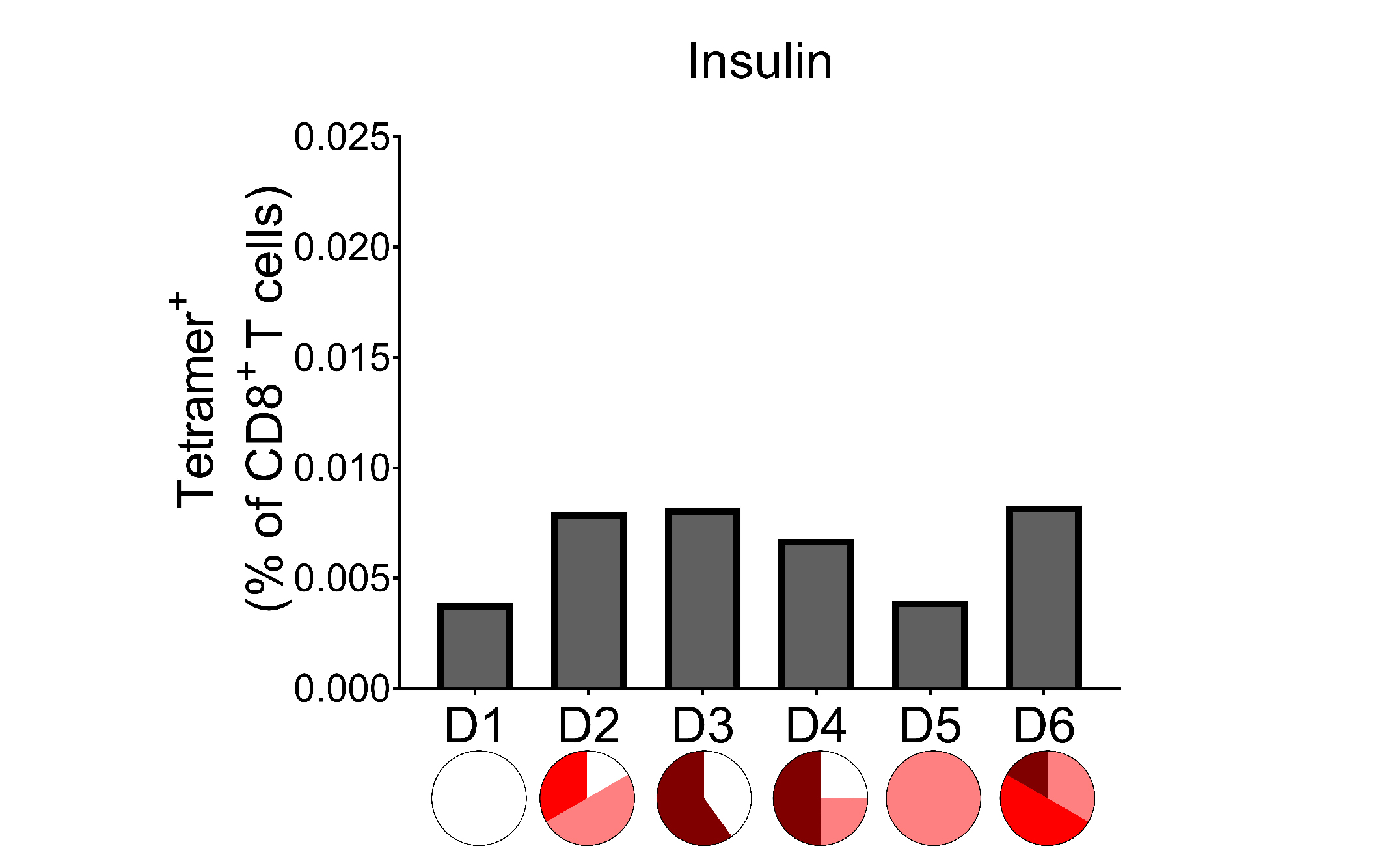


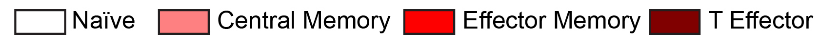


**Figure S3**. Insulin 10-18 peptide loaded tetramer (HLA-A2*02:01) staining of CD8+ T cells. Six HLA-A*02:01 positive donors CD8+ T cells were stained with CD45RA, CD45RA, and CCR7 directed antibodies to distinguish naïve, memory and effector subpopulations. Bars depict the tetramer positive cells among the CD8+ T cells and the pie charts show the proportion of each memory phenotype within the tetramer+ cells.


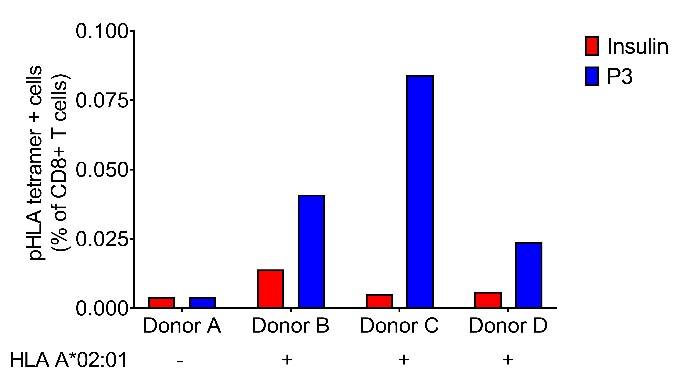

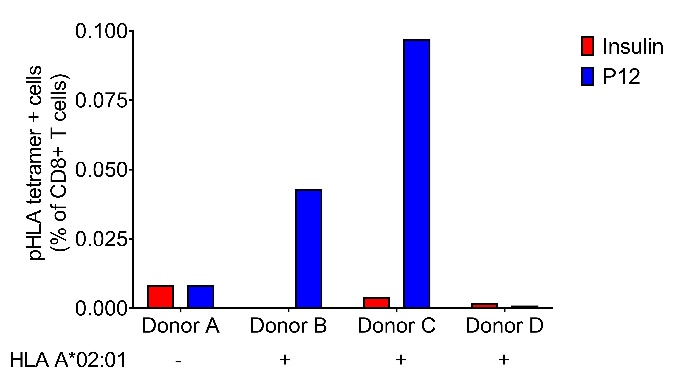

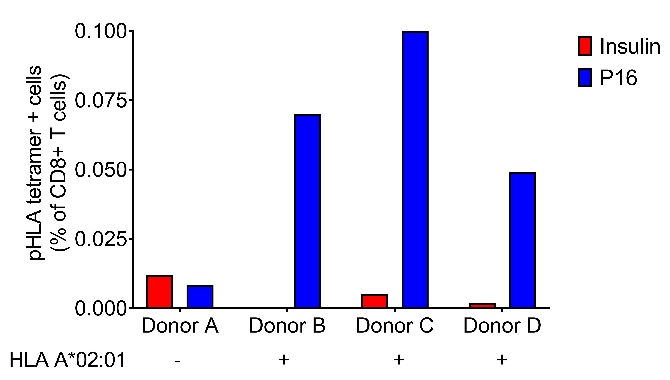

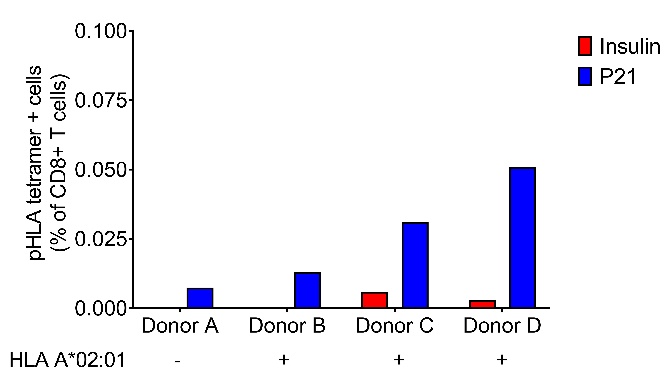


**Figure S4**. pHLA A*02:01-tetramer staining validation. PBMCs from one HLA A*02:01 negative and three HLA A*02:01 positive donors were stained anti-CD8-FITC, anti-CD3-PEcy7, insulin 10-18 loaded tetramers-PE and SARS-CoV-2 peptides loaded tetramers-APC. CD8+ T cells were analyzed after tetramers co-staining. Gating of the positive events was defined by using a FMO for each channel (either PE or APC). No double positive events (insulin tetramer-PE+ / P3-21 tetramer-APC+) were detected in any of the analyzed samples. Results are shown for one HLA-A*02:01 negative donor (Donor A) and three HLA-A*02:01 positive donors (Donors B, C and D). The donors used for this validation experiment were different from the ones used throughout the article.


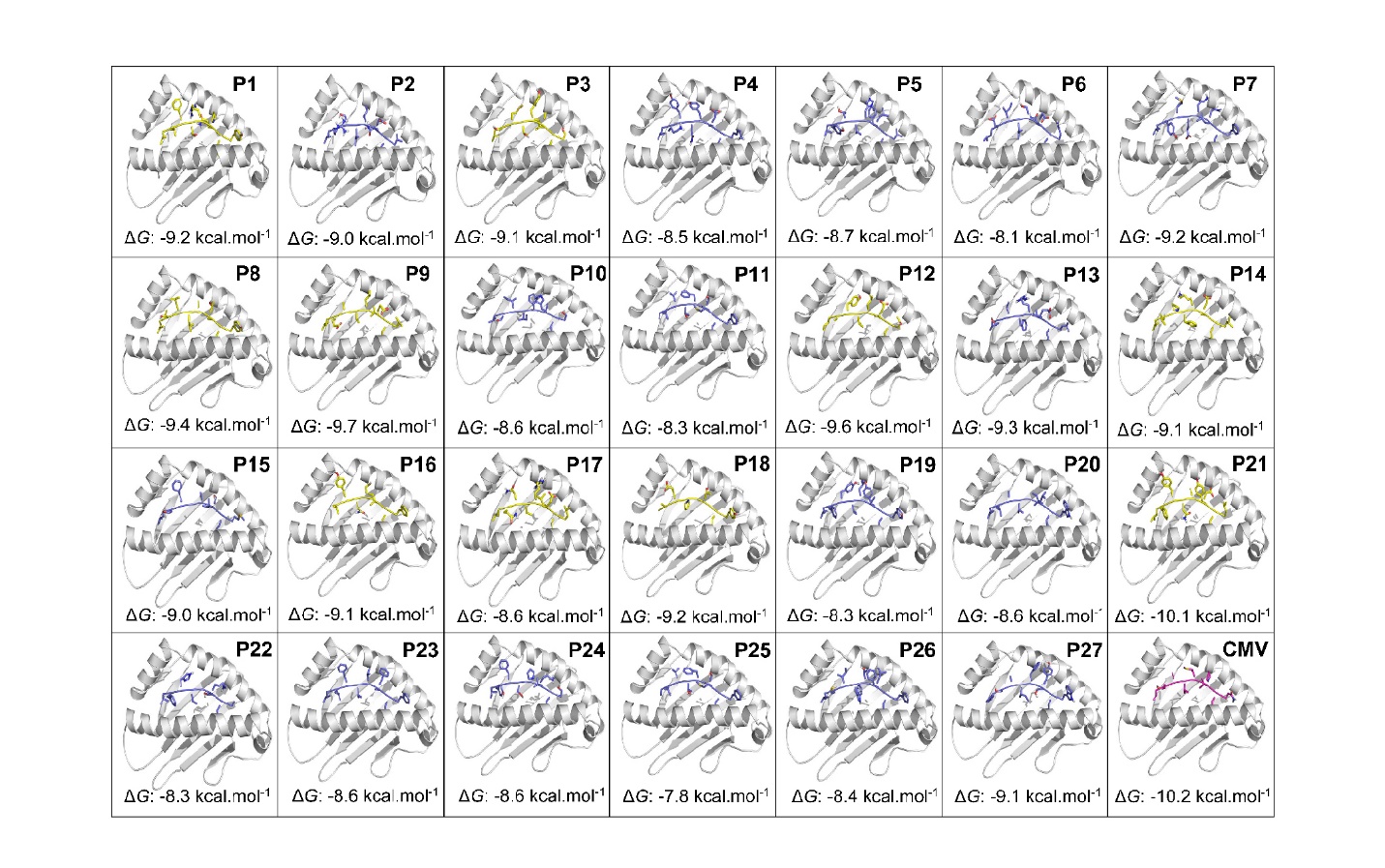


**Figure S5**. Docking analysis of HLA-A*02:01/epitope complexes. Selected and excluded peptides are shown in yellow and blue backbones, respectively. The CMV epitope in shown in magenta. HLA-A*02:01 (gray), only the epitope binding domain is illustrated. The binding energy was calculated for each peptide-HLA (pHLA) complex.

**Table S6**. Long synthetic peptides sequences. Eight aminoacids from the native protein sequence were added to each flank of the predicted epitope (highlighted in red). A nine arginine tail was added in the C-terminal as a cell penetrating domain to facilitate cellular entry.

| **Peptide** | **Long synthetic peptide sequence** |
| --- | --- |
| P3 | RARSVASQSIIAYTMSLGAENSVAYRRRRRRRRR |
| P12 | SRQRLTKYTMADLVYALRHFDEGNCRRRRRRRRR |
| P16 | NVNASSSEAFLIGCNYLGKPREQIDRRRRRRRRR |
| P21 | WKCRSKNPLLYDANYFLCWHTNCYDRRRRRRRRR |


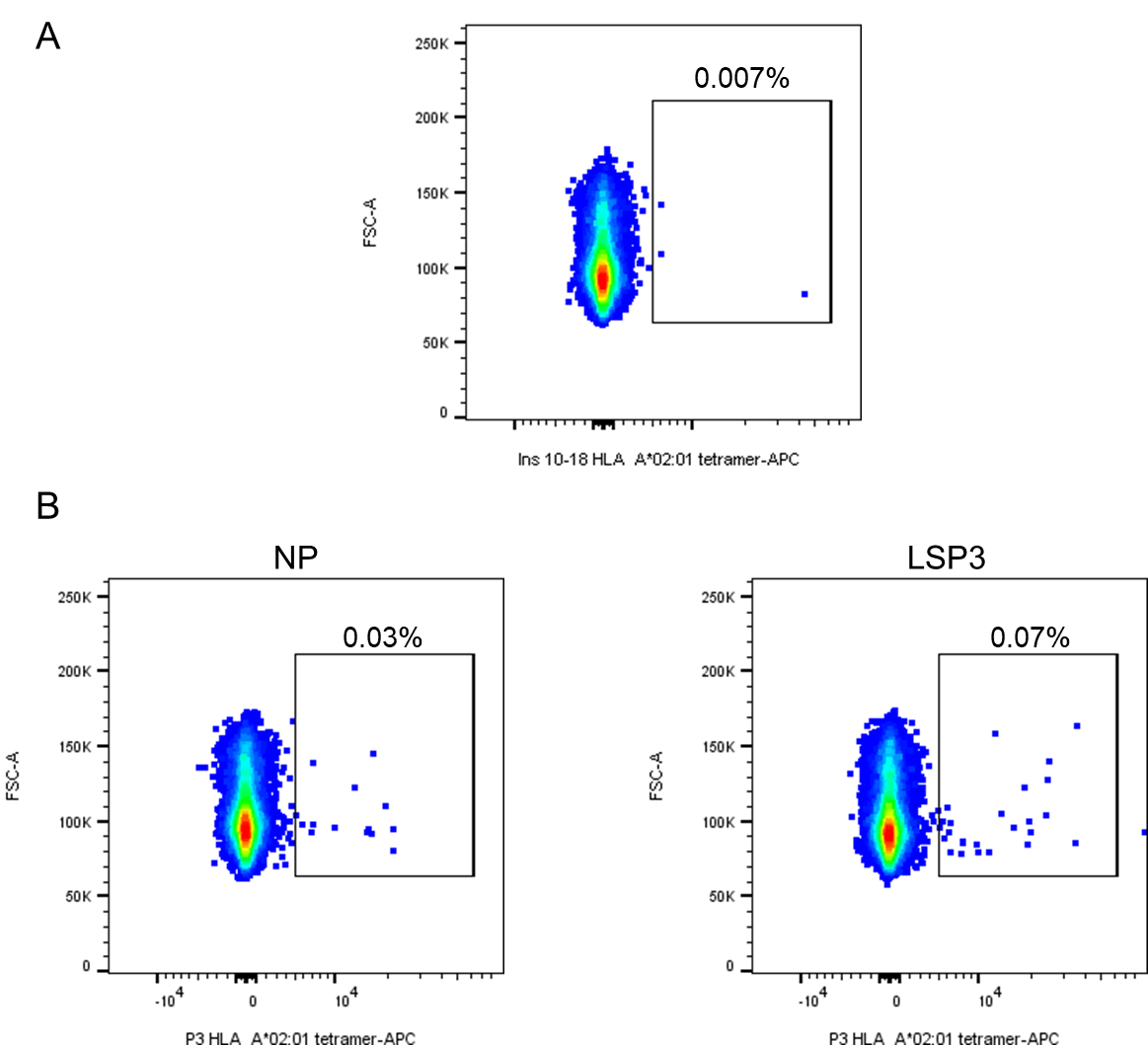


**Figure S7**. Representative figure for the gating of pHLA A*02:01 tetramer+ cells after LSP3 stimulation of CD8+ T cells. After a one week stimulation of PBMCs with either no peptide (NP) or LSP3 loaded dendritic cells, cells were retrieved for HLA A*02:01 tetramer APC staining. (**a**) To compensate for unspecific binding to tetramers, the PBMCs were stained using HLA A*02:01 tetramers loaded with insulin 10-18. (**b**) Representative results for the stimulated CD8+ T cells from donor 10 (D10) stained with the P3 HLA A*02:01 tetramers. The LSP3 stimulated cells show a 2.3 fold expansion in terms of P3 HLA A*02:01 tetramer + CD8+ T cells.

**Table S8**. Single CDR3 frequencies in the MIRA database (top 60 most frequent clones) for each of the peptide pools containing the selected peptides. Patients were filter based on HLA-A*02:01 expression, each time a CDR3 appeared was considered as a hit.

| **P3 (CDR3)** | **# of hits** | **P12 (CDR3)** | **# of hits** | **P21 (CDR3)** | **# of hits** |
| --- | --- | --- | --- | --- | --- |
| CASSSYNEQFF | 25 | CASSVGTSGYNEQFF | 14 | CASSLGGTEAFF | 59 |
| CASSLSGNQPQHF | 18 | CASSPQGTEAFF | 11 | CASSLGGAEAFF | 18 |
| CASSSTDTQYF | 15 | CASSQGVYEQYF | 11 | CASSLGETQYF | 16 |
| CASSLLGDTQYF | 13 | CSARDLAGSSYEQYF | 9 | CASSLGGSEAFF | 14 |
| CASSFGGNQPQHF | 12 | CASSLTGGDTEAFF | 7 | CASSLGGSTEAFF | 13 |
| CASSLRGGYEQYF | 11 | CASSQGLYEQYF | 7 | CASSLGGNEQFF | 13 |
| CASSFGGSYEQYF | 10 | CASSLEGGSYEQYF | 6 | CASSLGTGGTEAFF | 13 |
| CASSITGNQPQHF | 9 | CASSQGTYEQYF | 5 | CSARQGYEQYF | 12 |
| CASSSGLAYEQYF | 8 | CASSRGTYEQYF | 5 | CASSLGQETQYF | 10 |
| CASSLGGREQYF | 6 | CSASQGTYEQYF | 5 | CASSLGSTEAFF | 9 |
| CASSLSDTQYF | 6 | CASRQGGNQPQHF | 3 | CASSVAGSTEAFF | 9 |
| CASSPTGNTGELFF | 6 | CASSLGRQETQYF | 3 | CATSRDPGSNQPQHF | 8 |
| CASLGETQYF | 5 | CASSLGTGYTEAFF | 3 | CSARGTGGTEAFF | 8 |
| CASSSGLSYEQYF | 5 | CASSLSGSGETQYF | 3 | CASSQGSYEQYF | 6 |
| CASSSQGQPQHF | 5 | CASSQGTGPYEQYF | 3 | CASSLEGYEQYF | 6 |
| CASSGGYEQYF | 4 | CASSYSDSYEQYF | 3 | CASSLTGTNEKLFF | 6 |
| CASSIPTEAFF | 4 | CSARAGEQFF | 3 | CASSIDGSYEQYF | 5 |
| CASSLGGITEAFF | 4 | CSARGTSGNTGELFF | 3 | CASSPGTGGDTQYF | 5 |
| CASSLGTGGYEQYF | 4 | CSARQGITEAFF | 3 | CASSFGGAEAFF | 5 |
| CASSLTGAYNEQFF | 4 | CASRQGYNEQFF | 2 | CSVEGTEAFF | 5 |
| CASSVDSNSPLHF | 4 | CASSLGLYEQYF | 2 | CASSLGSGGTEAFF | 5 |
| CSAPGGYEQYF | 4 | CASSLSGGGEKLFF | 2 | CASSLQGSNQPQHF | 4 |
| CASFGDTQYF | 3 | CASSLSGQGYEQYF | 2 | CASSLGTGSYEQYF | 4 |
| CASFGDTQYF | 3 | CASSPRVNMNTEAFF | 2 | CASSVGDNSPLHF | 4 |
| CASSFDTGELFF | 3 | CASSPTVNMNTEAFF | 2 | CSAREIQETQYF | 4 |
| CASSFGAGELFF | 3 | CASSQGGYNEQFF | 2 | CASSLAGSTEAFF | 4 |
| CASSFGGPYEQYF | 3 | CSAKAGTDTQYF | 2 | CASRDRDYEQYF | 3 |
| CASSLGGPEAFF | 3 | CSANRGTYEQYF | 2 | CSARQNTGELFF | 3 |
| CASSLGRGYEQYF | 3 | CAISARGGQGTYEQYF | 1 | CASSYGGANSPLHF | 3 |
| CASSLLGTGGYNEQFF | 3 | CASLSGNTIYF | 1 | CASSLSYTGELFF | 3 |
| CASSLQGLNTEAFF | 3 | CASSFGDTQYF | 1 | CASSEGQIYEQYF | 3 |
| CASSQGGGTDTQYF | 3 | CASSLAGGEQYF | 1 | CASSRQGSYNEQFF | 3 |
| CASSQYNEQFF | 3 | CASSLAPEPREPQHF | 1 | CASSLGGLGYTF | 3 |
| CASSSGYNEQFF | 3 | CASSLGAGYTEAFF | 1 | CASSRVSNQPQHF | 3 |
| CASSSNTDTQYF | 3 | CASSLGGYNEQFF | 1 | CSVVREGYEQYF | 2 |
| CASSSQGNTEAFF | 3 | CASSLTDNQPQHF | 1 | CASSYTNEQFF | 2 |
| CASSSSLVYEQFF | 3 | CASSPGGYNEQFF | 1 | CSASRTGPYEQYF | 2 |
| CASGLQYEQYF | 2 | CASSPGGYTEAFF | 1 | CASSWDRSSYEQYF | 2 |
| CASGTRGNEQFF | 2 | CASSPGPNTEAFF | 1 | CASRGQGAYGYTF | 2 |
| CASHGDTQYF | 2 | CASSPLGSSYNSPLHF | 1 | CASSLLQGSNQPQHF | 2 |
| CASLGDTQYF | 2 | CASSPRDRSYNEQFF | 1 | CSARAGGYNEQFF | 2 |
| CASRSEGNQPQHF | 2 | CASSPWVNRNTEAFF | 1 | CASSLDGAEAFF | 2 |
| CASSFGGVNEQFF | 2 | CASSQGLAPYNEQFF | 1 | CSARAENTEAFF | 2 |
| CASSIGQGTDTQYF | 2 | CASSSSVSSYNEQFF | 1 | CSARGTNTGELFF | 2 |
| CASSK*GANQPQHF | 2 | CASSVGVGGYNEQFF | 1 | CASSLLQGNQPQHF | 2 |
| CASSLASGYNEQFF | 2 | CASSWTGRGETQYF | 1 | CASSPNSYNEQFF | 2 |
| CASSLATAYGYTF | 2 | CASSYFSTDTQYF | 1 | CASSWTASNQPQHF | 2 |
| CASSLEGSEAFF | 2 | CASTPGQGSYEQYF | 1 | CASSYSSVYEQYF | 2 |
| CASSLEGVNQPQHF | 2 | CASTVQGYTEAFF | 1 | CASSLGQGAQPQHF | 2 |
| CASSLFGNTEAFF | 2 | CATLEGSYNEQFF | 1 | CSARLANTGELFF | 2 |
| CASSLGGKTQYF | 2 | CATSDPRQGAYNEQFF | 1 | CASSGQGNSPLHF | 2 |
| CASSLGGNYEQYF | 2 | CATSEVGSTEAFF | 1 | CASSEGGQPQHF | 2 |
| CASSLGGPEQYF | 2 | CSAGDRGKGETQYF | 1 | CASSLTGGDTQYF | 2 |
| CASSLLGETQYF | 2 | CSARDIGVEAFF | 1 | CASSLGGTGAFF | 2 |
| CASSLLGTGELFF | 2 | CSASEGTYEQYF | 1 | CSAGYTSGRAVFYNEQFF | 2 |
| CASSLSLAGADTQYF | 2 | CSASLGAYEQYF | 1 | CASSLSGSTEAFF | 2 |
| CASSLVGVYEQYF | 2 | CSASLGVYEQYF | 1 | CASSTTGTRGSNEQFF | 2 |
| CASSPGLANEQFF | 2 | CSASQGAYEQYF | 1 | CASSRTGGNQPQHF | 2 |
| CASSPGRGNQPQHF | 2 | CSASQGLYEQYF | 1 | CASSLGTGGHQETQYF | 1 |
| CASSPNMFEAFF | 2 | CSASRGLYEQYF | 1 | CASSAGTGGGETQYF | 1 |

**Table S9**. Proportion of patients within the MIRA database with the following SARS-CoV-2 reactive T cell clonotypes. Proportions are shown for the top three most frequent reactive clonotypes for each of the peptide pools containing the P3, P12 or P21 peptides. 31 COVID-19 HLA-A*02:01+ patients were considered for this analysis.

| **P3 (CDR3)** | **% donors** | **P12 (CDR3)** | **% donors** | **P21 (CDR3)** | **% donors** |
| --- | --- | --- | --- | --- | --- |
| CASSSYNEQFF | 45.16 | CASSVGTSGYNEQFF | 32.25 | CASSLGGTEAFF | 51.61 |
| CASSLSGNQPQHF | 41.93 | CASSPQGTEAFF | 22.58 | CASSLGGAEAFF | 29.03 |
| CASSSTDTQYF | 32.25 | CASSQGVYEQYF | 25.80 | CASSLGETQYF | 38.71 |


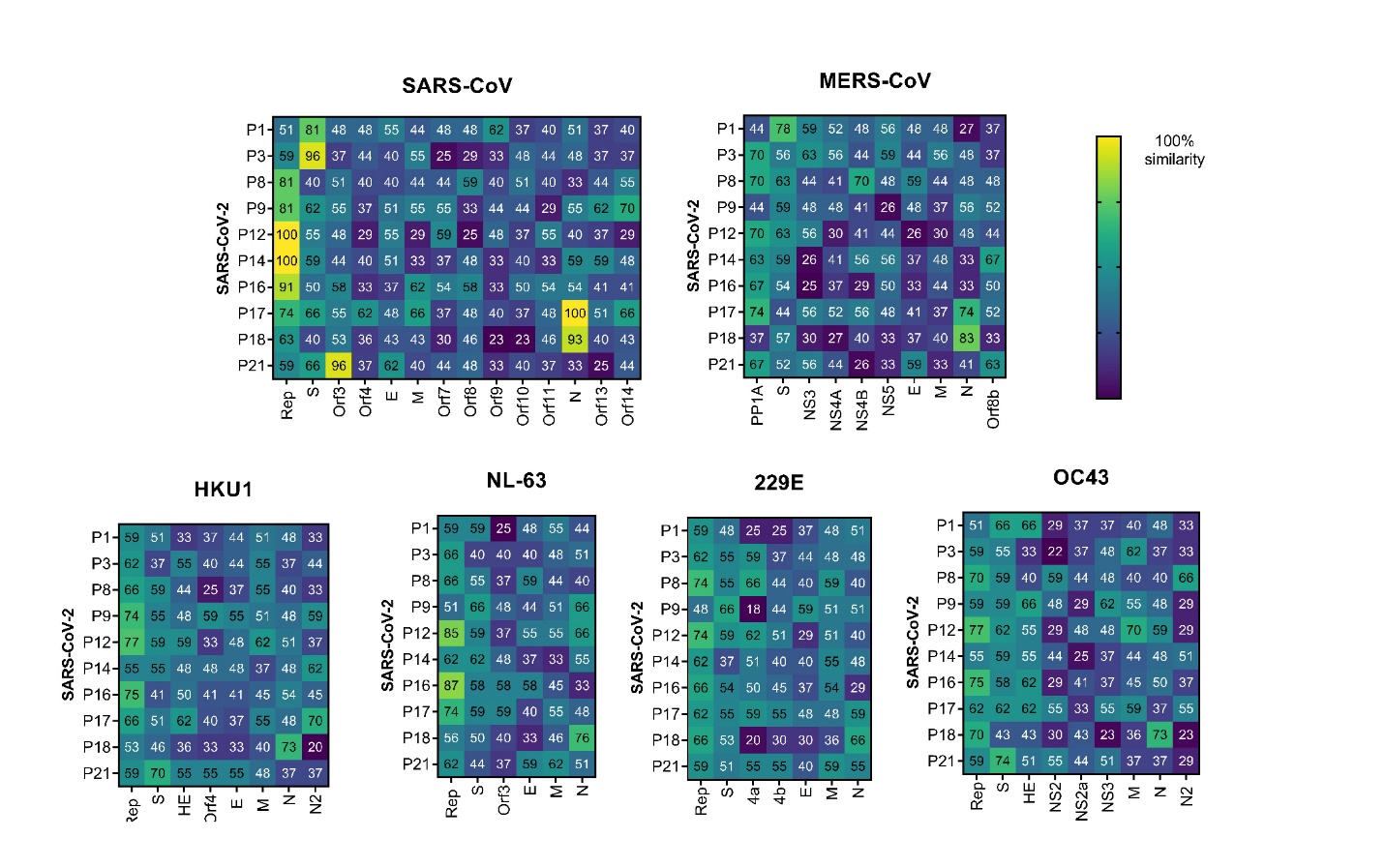


**Figure S10**. Heatmap representation of a similarity matrix between SARS-CoV-2 epitopes and other human infecting coronaviruses. Each SARS-CoV-2 predicted epitope was separately aligned with each protein of other coronaviruses including SARS-CoV, MERS-CoV, OC43, HKU1, NL-63, and 229E. The similarity percentage between each pairs are indicated in numbers. Yellow and blue colors refer to the higher and lower similarity, respectively.
